# A Stable Thoracic *Hox* Code and Epimorphosis Characterize Posterior Regeneration in *Capitella teleta*

**DOI:** 10.1371/journal.pone.0149724

**Published:** 2016-02-19

**Authors:** Danielle M. de Jong, Elaine C. Seaver

**Affiliations:** Whitney Laboratory for Marine Bioscience, University of Florida, St. Augustine, Florida, United States of America; Laboratoire de Biologie du Développement de Villefranche-sur-Mer, FRANCE

## Abstract

Regeneration, the ability to replace lost tissues and body parts following traumatic injury, occurs widely throughout the animal tree of life. Regeneration occurs either by remodeling of pre-existing tissues, through addition of new cells by cell division, or a combination of both. We describe a staging system for posterior regeneration in the annelid, *Capitella teleta*, and use the *C*. *teleta Hox* gene code as markers of regional identity for regenerating tissue along the anterior-posterior axis. Following amputation of different posterior regions of the animal, a blastema forms and by two days, proliferating cells are detected by EdU incorporation, demonstrating that epimorphosis occurs during posterior regeneration of *C*. *teleta*. Neurites rapidly extend into the blastema, and gradually become organized into discrete nerves before new ganglia appear approximately seven days after amputation. *In situ* hybridization shows that seven of the ten *Hox* genes examined are expressed in the blastema, suggesting roles in patterning the newly forming tissue, although neither spatial nor temporal co-linearity was detected. We hypothesized that following amputation, *Hox* gene expression in pre-existing segments would be re-organized to scale, and the remaining fragment would express the complete suite of *Hox* genes. Surprisingly, most *Hox* genes display stable expression patterns in the ganglia of pre-existing tissue following amputation at multiple axial positions, indicating general stability of segmental identity. However, the three *Hox* genes, *CapI-lox4*, *CapI-lox2* and *CapI-Post2*, each shift its anterior expression boundary by one segment, and each shift includes a subset of cells in the ganglia. This expression shift depends upon the axial position of the amputation. In *C*. *teleta*, thoracic segments exhibit stable positional identity with limited morphallaxis, in contrast with the extensive body remodeling that occurs during regeneration of some other annelids, planarians and acoel flatworms.

## Introduction

Regeneration, the ability to replace lost tissues and body parts following traumatic injury, is present in representatives of most metazoan phyla [1,2]. In some animals, this ability is limited to the replacement of a particular cell type, tissue or structure (e.g. the limb of axolotls, the fins of zebrafish), while others can rebuild their entire bodies from a single piece of tissue (e.g. planarians, cnidarians). Although variation in regeneration ability exists, the widespread phylogenetic distribution of some form of regenerative capability in the Metazoa suggests an evolutionary ancient origin of regeneration with subsequent multiple losses across many lineages.

To enable comparisons when discussing regeneration in diverse animals, T.H. Morgan coined the terms epimorphosis and morphallaxis. Epimorphosis occurs when cell proliferation leads to formation of new tissue, while morphallaxis is characterized by re-patterning of existing tissue, in the absence of cell proliferation [3]. Epimorphic regeneration can involve the production of a blastema, a group of cells of variable potency that appear at the wound site following wound healing. Much of the current regeneration research is devoted to studies of epimorphosis, and focuses on identifying the source of cells that form the regenerated tissue, and determining the mechanisms by which these cells regenerate the appropriate tissues (reviewed in [2,4]). Morphallaxis is primarily known from invertebrate species, and a number of striking cases have been described. For example, to reconstitute the original complement of tissues along the main body axis following transverse amputation, *Hydra* respecifies and rearranges the remaining tissue (reviewed in [5]), the oligochaete annelid *Enchytraeus* respecifies its gut[6], the sabellid annelid, *Sabella* changes identity of some of its segments [7], the acoel *Hofstenia* regains its transverse pigment stripes [8] and planarians reform their pharynx (reviewed in [9]). The broad phylogenetic distribution of documented examples of morphallaxis suggests that this might be a more widespread phenomenon than previously appreciated.

Annelids are the segmented worms, and have long been studied as models of regeneration, due to their impressive regenerative capabilities. Most species display robust posterior regeneration, a trait that is likely ancestral for annelids [10]. Some species that can regenerate posterior tissues can also regenerate their anterior segments and head. Regeneration in many annelids involves a combination of both epimorphosis (cell proliferation resulting in formation of new tissue) and morphallaxis (reorganization of pre-existing tissue) (e.g. [6,11,12]).

The annelid *Capitella teleta* can regenerate posterior but not anterior segments following transverse amputation [13,14]. There is little published data on whether posterior regeneration in *C*. *teleta* proceeds via epimorphosis, morphallaxis or a combination of the two. A blastema forms within 1–3 days following transverse amputation of adults [14], however thus far, morphallaxis during regeneration of *C*. *teleta* has not been reported. *C*. *teleta* has a number of morphological features uniquely distributed along the anterior-posterior axis that facilitate studies of patterning during regeneration. There are two distinct body regions composed of nine thoracic segments, and a variable number of posterior abdominal segments. In addition, many segments have unique structures. For example, male reproductive organs are present in segments 7 and 8, and female reproductive organs are present in most abdominal segments [15], the gut is highly regionalized along its length (pharynx, esophagus, midgut and hindgut) [16], a pair of FMRFamide immunoreactive neurons are restricted to segment 5 [17], and there are distinct chaetal morphologies between the thoracic and abdominal segments (simple or hooded hook chaetae, respectively) [18].

**Fig 1:**
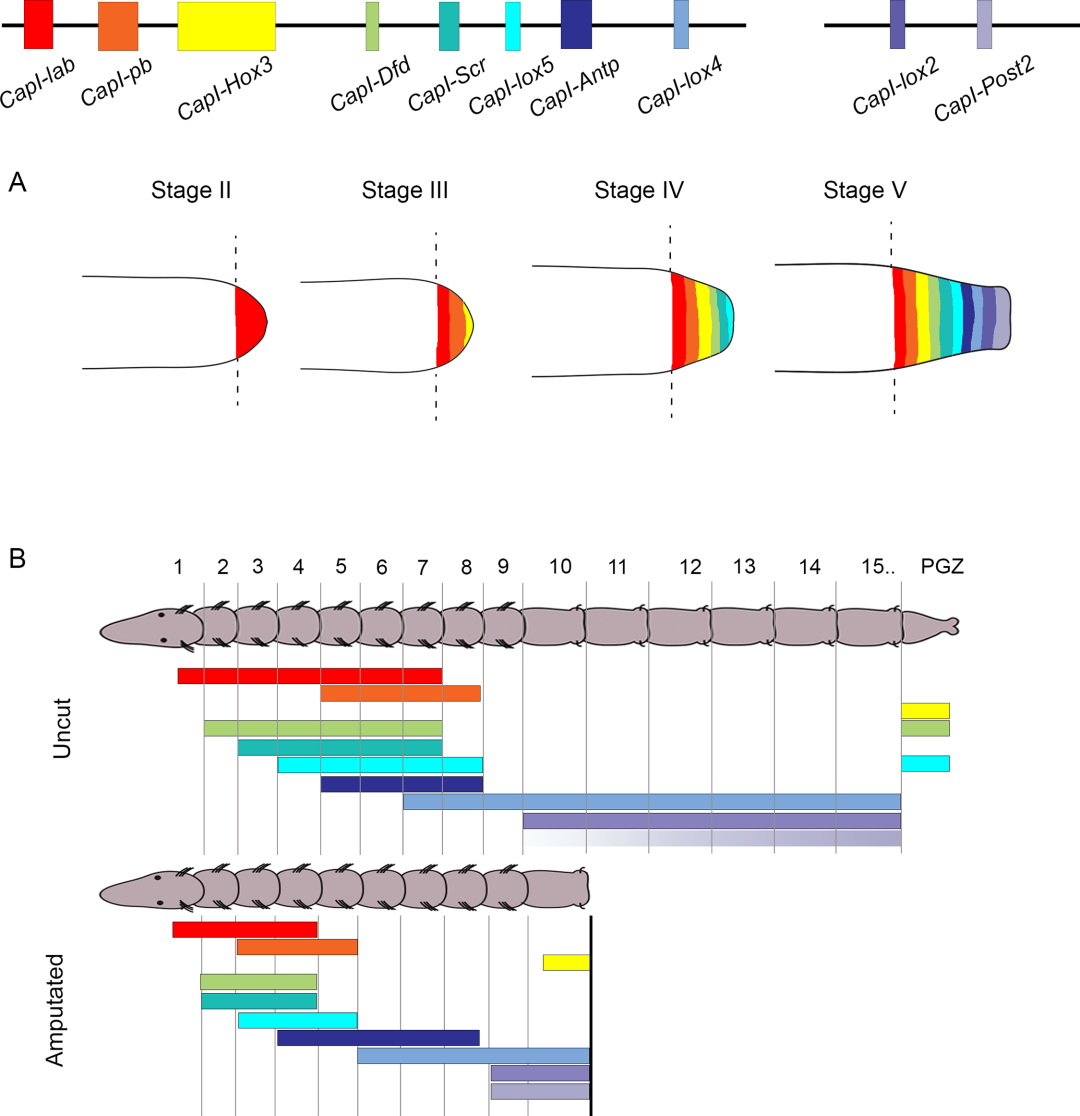
Schematic representation of hypothesized *Hox* gene expression patterns in the blastema and pre-existing tissue during regeneration. At the top of the figure, black lines depict two scaffolds that contain 10 of the *C*. *teleta Hox* genes. Colored rectangles depict the coding sequence of each *Hox* gene. Color coding in (A) and (B) is the same as that used in the schematic depicting the genomic organization of *Hox* genes. (A) Schematic representation of the posterior end of regenerating juveniles at Stage II, III, IV and V of regeneration. Black dotted lines depict the amputation site. Colored areas within the blastema indicate regions of gene expression, with each color corresponding to a different *Hox* gene. If *C*. *teleta Hox* genes exhibited spatial co-linearity in the blastema during regeneration, genes at one end of the cluster (e.g. *CapI-lab*; see red band depicting expression at Stage V) would be expressed more anteriorly in the blastema than genes at the other end of the cluster (e.g. *CapI-Post2*; see light purple band depicting expression at Stage V). If the *C*. *teleta Hox* genes exhibited temporal co-linearity in the blastema during regeneration, genes at one end of the cluster would be expressed earlier in regeneration (e.g. *CapI-lab* expression initiated at Stage II) than genes at the other end of the cluster (e.g. *CapI-Post2* expression initiated at Stage V). (B) Schematic representation of a juvenile of 15 segments and posterior growth zone (PGZ), oriented with anterior to the left. Colored bars indicate expression of *Hox* genes along the anterior-posterior axis. In uncut animals, the *Hox* genes have defined anterior and posterior boundaries of expression, with staggered anterior boundaries. The anterior boundary of *CapI-Post2* is variable, as indicated by a purple gradient. In unamputated animals, if morphallaxis occurred during posterior regeneration in *C*. *teleta*, we hypothesize there would be an anterior shift in expression of each *Hox* gene in the remaining segments.

A previous study of *C*. *teleta Hox* genes–a family of transcription factors that define regional identities along the anterior-posterior axis during development in a wide variety of animals–revealed molecular differences along the anterior-posterior axis [19]. In *C*. *teleta*, there are 11 distinct paralogous groups, each containing a single gene, and at least 8 *Hox* genes are linked in a single cluster within the genome. *Hox* genes in *C*. *teleta* larvae are expressed in broad ectodermal domains, and in juveniles most *Hox* genes are expressed specifically in the ganglia of the ventral nerve cord and in the posterior growth zone. Most genes have stable, discrete anterior and posterior expression boundaries, and exhibit spatial and temporal colinearity, with staggered anterior boundaries in the thoracic and anterior abdominal segments ([19]; see Fig 1B). In the nine thoracic segments of juveniles, there are eight unique combinations of *Hox* genes expressed, suggesting that almost every thoracic segment has a unique molecular identity. In contrast, all abdominal segments express the same three *Hox* genes.

We hypothesized that both epimorphosis and morphallaxis occur during posterior regeneration in *C*. *teleta*. If epimorphosis occurs, we would expect cell proliferation to contribute to the formation of the regeneration blastema. Furthermore, if *Hox* genes are involved in anterior-posterior patterning of the blastema, we propose that they will be expressed in staggered domains with temporal and spatial co-linear expression characteristics during its outgrowth (Fig 1A). For example, following amputation, we would expect *CapI-pb*, which is positioned at one end of the cluster, to be expressed earlier and in a more anterior location than *CapI-Post2*, which is positioned at the other end of the *Hox* cluster. If morphallaxis occurs in *C*. *teleta*, we hypothesize that amputation of a posterior piece of the animal would result in an anterior shift in expression of *Hox* genes in the pre-existing tissue (Fig 1B). These shifts would serve to re-establish all positional identities in the remaining tissue in an animal with a reduced number of segments.

We investigated the dynamics of regeneration following transverse amputation at various axial positions in *C*. *teleta* juveniles at 2 weeks post-metamorphosis, a stage at which juveniles have approximately 22–28 segments. Analysis of *Hox* gene expression in the context of regeneration allowed us to examine two distinct aspects of regeneration: morphallaxis of pre-existing tissue, and patterning in the blastema. To facilitate our analyses, we developed a staging system to describe the first 7 days of posterior regeneration. We describe changes in nervous system innervation and gut ciliation, reformation of the pygidium and posterior growth zone (PGZ), and quantify cell proliferation by EdU incorporation. Our results demonstrate that epimorphosis occurs during posterior regeneration of *C*. *teleta*, resulting in the formation of a regeneration blastema. Seven out of ten *Hox* genes are expressed within the blastema at different stages of its outgrowth. However, they do not exhibit spatial or temporal co-linearity, suggesting that they do not play a role in anterior-posterior patterning of the blastema. We also examined *Hox* gene expression in pre-existing tissue following amputation at multiple axial positions. Specifically, animals were amputated between segments 7 and 11, within the expression domains of *CapI-pb*, *CapI-Scr*, *CapI-lox5*, *CapI-Antp*, *CapI-lox4*, *CapI-lox2* and *CapI-Post2*, and the expression pattern of each *Hox* gene in pre-existing segments was compared to uncut controls. Most *Hox* genes have stable expression patterns in pre-existing tissue following removal of posterior pieces of the animal. However, three *Hox* genes (*CapI-lox2*, *CapI-lox4* and *CapI-Post2*) shift their expression boundary in an anterior direction in pre-existing segments, and these shifts depend upon the axial position of amputation. The shift in expression of *CapI-lox2* and *CapI-lox4* includes a subset of cells in the ganglia. Our results demonstrate that segments exhibit a stable identity, and only limited morphallaxis occurs during *C*. *teleta* regeneration.

## Materials and Methods

### Cloning of *C*. *teleta CapI-Hox3*, *CapI-lox4* and *CapI-lox5* genes

Although a previous publication reported the cloning and expression patterns of all *Hox* genes also used in this study [19], it was necessary to reclone three *Hox* genes. We verified that these clones produced expression patterns identical to previously published results. Primer design was based on transcript sequences previously submitted to GenBank (*CapI-Hox3* EU196539; *CapI-lox4* EU196543; *CapI-lox5* EU196542). Amplified fragments were 1283 bp (*CapI-Hox3*), 983 bp (*CapI-lox4*) or 1506 bp (*CapI-lox5*) and were cloned into pGEM-T_easy_ vector (Promega) before sequencing at the University of Hawaii or Macrogen Corp (Maryland).

### Animal husbandry and amputations

A *C*. *teleta* colony was maintained in the laboratory at 19°C according to published culture methods [20]. Larvae were allowed to emerge naturally from the maternal brood tube, and animals were maintained in bowls with filtered seawater (FSW) and previously frozen and sieved estuarine mud as a food source. Regeneration experiments were performed on juveniles at 2 weeks post-metamorphosis. Animals were removed from the mud and placed in 0.5% cornmeal agar:FSW plates supplemented with 60 μg/mL penicillin plus 50 μg/mL streptomycin. Prior to amputation, animals were immobilized in 0.5% cornmeal agar supplemented with MgCl_2_ (1:1 FSW:0.37 M MgCl_2_) for a maximum of 30 minutes, and then placed in a drop of 0.37 M MgCl_2_:FSW (1:1) on a platform of black dissecting wax (American Educational Products). Animals were amputated at the posterior edge of the target segment using a microsurgery scalpel (Feather; 15 degree blade), and then returned to 0.5% cornmeal agar:FSW dishes for up to 24 hours. For analysis of longer periods of regeneration, animals were then placed into bowls of FSW and sieved estuarine mud for the desired length of time. Prior to fixation, animals were recovered from mud and placed in 0.5% cornmeal agar:FSW dishes for 1–6 hours to remove mud and debris, immobilized in 0.5% cornmeal agar with MgCl_2_ for 15 minutes, and fixed in either 3.7% paraformaldehyde:FSW at 4°C for 16 to 24 h for *in situ* hybridization, or at room temperature for at least 30 minutes for immunohistochemistry. Following fixation, animals were processed for either whole-mount *in situ* hybridization or immunohistochemistry (see below).

### Whole mount *in situ* hybridization

Following fixation, intact and regenerating juveniles were washed in phosphate-buffered saline (PBS), dehydrated through a methanol series to 100% methanol, and stored at -20°C for up to 4 weeks. Digoxigenin-labeled riboprobes for all genes were generated with the SP6 or T7 MEGAscript kit (Ambion, Inc., Austin, TX, USA). Probe lengths and RNA polymerase used are as follows; *CapI-Post2*, 760 bp, SP6; *CapI*-*lox2*, 780 bp, SP6; *CapI-lox4*, 983 bp, SP6; *CapI-Antp*, 1600 bp, SP6; *CapI-lox5*, 1506 bp, SP6; *CapI*-*Scr*, 900 bp, T7; *CapI*-*Dfd*, 630 bp, T7; *CapI-pb*, 800 bp, SP6; *CapI-lab*, 800 bp, T7; *CapI-Hox3*, 1292 bp, SP6. Probes were diluted to a final concentration of 1 ng/μL. Whole-mount *in situ* hybridization followed published protocols [21]. Following hybridization at 65°C for 48–72 h, probes were detected using nitro blue tetrazolium chloride/5-bromo-4-chloro-3-indolyphosphate (NBT/BCIP) color substrate. Typically, the color reaction was allowed to develop between three hours and three days. Specimens were equilibrated in glycerol (80% glycerol:1x PBS) and placed on Rainex-coated slides. At least 2 independent repetitions were performed for each experiment, and typically gene expression patterns were analyzed for at least 10–15 individuals. The expression patterns described represent the majority of cases. In juveniles, the 2 to 3 anterior-most ganglia are out of register with segmental boundaries and straddle adjacent segments. We report expression as it corresponds to ganglion number, with the anterior-most ganglia denoted as ganglion 1.

### Detection of cell proliferation

The Click-iT EdU Alexa Fluor 488 Imaging Kit (Life technologies C10337) was used to label cells undergoing DNA synthesis, following the manufacturer recommendations. Briefly, juveniles were exposed to 5’-ethynyl-2’-deoxyuridine (EdU) at a final concentration of 3 μM for 1 hour or 8 hours, depending upon the experiment. Animals were then placed in 1:1 0.37 M MgCl_2_:FSW for 15 minutes before fixation overnight at 4°C in 3.7% paraformaldehyde:FSW. For some experiments, *in situ* hybridization was performed on samples prior to detection of EdU. Briefly, animals were rinsed several times with PBS and exposed to PBS+0.5% Triton-X100 before the EdU detection reaction was performed, following the manufacturer recommendations. Following the EdU detection reaction, antibody labeling was performed as described below.

### Immunohistochemistry

Following EdU incorporation and detection, juveniles were washed several times in PBS + 0.1% Triton (PBT), before being treated with block solution (PBT + 10% normal goat serum, Sigma G9023) for 45–60 minutes. Mouse anti-acetylated α-tubulin antibody (Sigma T6743) was diluted to 1:300 in block solution, and animals were incubated for 12–18 hours at 4°C. The following day, animals were washed twice in PBT, followed by four PBT washes of 20–30 minutes each. Donkey anti-mouse-546 secondary antibody (Invitrogen A21203) was diluted to 1:250 in block solution, and animals were incubated for 2–4 hours at room temperature. Following two rinses in PBT, four PBT washes of 20–30 minutes each were conducted. Finally, animals were equilibrated in 80% glycerol:PBS plus 0.125 μg/μL Hoechst 33342 (Life Technologies, H3570) overnight, before being imaged and analyzed as described below.

### Nuclei Counts and Statistical Analyses

To quantify EdU-positive cells, specimens were counterstained for total nuclei with Hoechst 33342, and then confocal z-stacks (see Microscopy section below) were rendered into 3D images and cropped according to which area of the animal was desired for analysis (8^th^, 9^th^, 10^th^ segment or regenerating tissue). Each z-stack was created from focal planes extending from the ventral ectoderm to the lumen of the gut. A segment was defined as the region of tissue extending from the posterior edge of the ganglion of the preceding segment, to the posterior edge of the ganglion of the segment of interest. Following amputation, new tissue was defined as tissue posterior to the posterior-most pre-existing ganglion (see S1A Fig). Once a particular area was defined, EdU-positive nuclei and total nuclei were digitally identified using Imaris Software (Bitplane, Switzerland), using a size threshold of 2uM, and a quality score of >15. Each area was examined to ensure digital identification of nuclei was accurate. The number of EdU-positive nuclei were divided by the total number of nuclei to generate a ratio for a particular area. Cell counts were taken from at least 5 individuals, and comparisons between different segments, or between segments and the newly formed tissue analyzed using a Student’s one-tailed *t*-test. Differences between samples with a *p* value <0.05 were considered statistically significant.

### Microscopy and Imaging

Specimens which had undergone *in situ* hybridization were imaged using an Axioskop 2 motplus compound microscope (Zeiss, Gottingen, Germany), coupled with a SPOT FLEX digital camera (Diagnostic Instruments, Inc., SterlingHeights, MI). Images were captured using SPOT imaging software and analyzed using Adobe Photoshop CS6 (version 13.0). Multiple DIC focal planes were merged for some images using Helicon Focus (Helicon Soft Ltd., Kharkov, Ukraine), as noted in the figure legends. Following immunohistochemistry or EdU labeling, animals were imaged using a Zeiss LSM 710 confocal microscope (Zeiss, Gottingen, Germany). Z-stack projections were generated using ImageJ (NIH). All figures were created in Adobe Photoshop CS6 (version 13.0).

## Results

### Posterior regeneration in *C*. *teleta*

A brief description of posterior regeneration in *C*. *teleta* has been published [14]; however, the previous study focused on reproductive adults (8 weeks post-metamorphosis), and here we provide a careful analysis of cell division patterns and blastema formation. To examine blastema formation, cell proliferation dynamics and ventral nerve cord architecture during posterior regeneration in *C*. *teleta*, we used nuclear staining, EdU incorporation and anti-acetylated α-tubulin reactivity, to compare the posterior end of 2 week old juveniles at various stages of regeneration following transverse amputation, with uncut controls. For quantification of proliferating cells, the ratio of EdU-positive nuclei to total nuclei were calculated in the three segments closest to the amputation site (segments 8, 9 and 10), at each stage of regeneration. The ratios for each segment were compared to the ratio of EdU-positive to total nuclei in the corresponding segment in uncut controls (S1A Fig).

**Fig 2:**
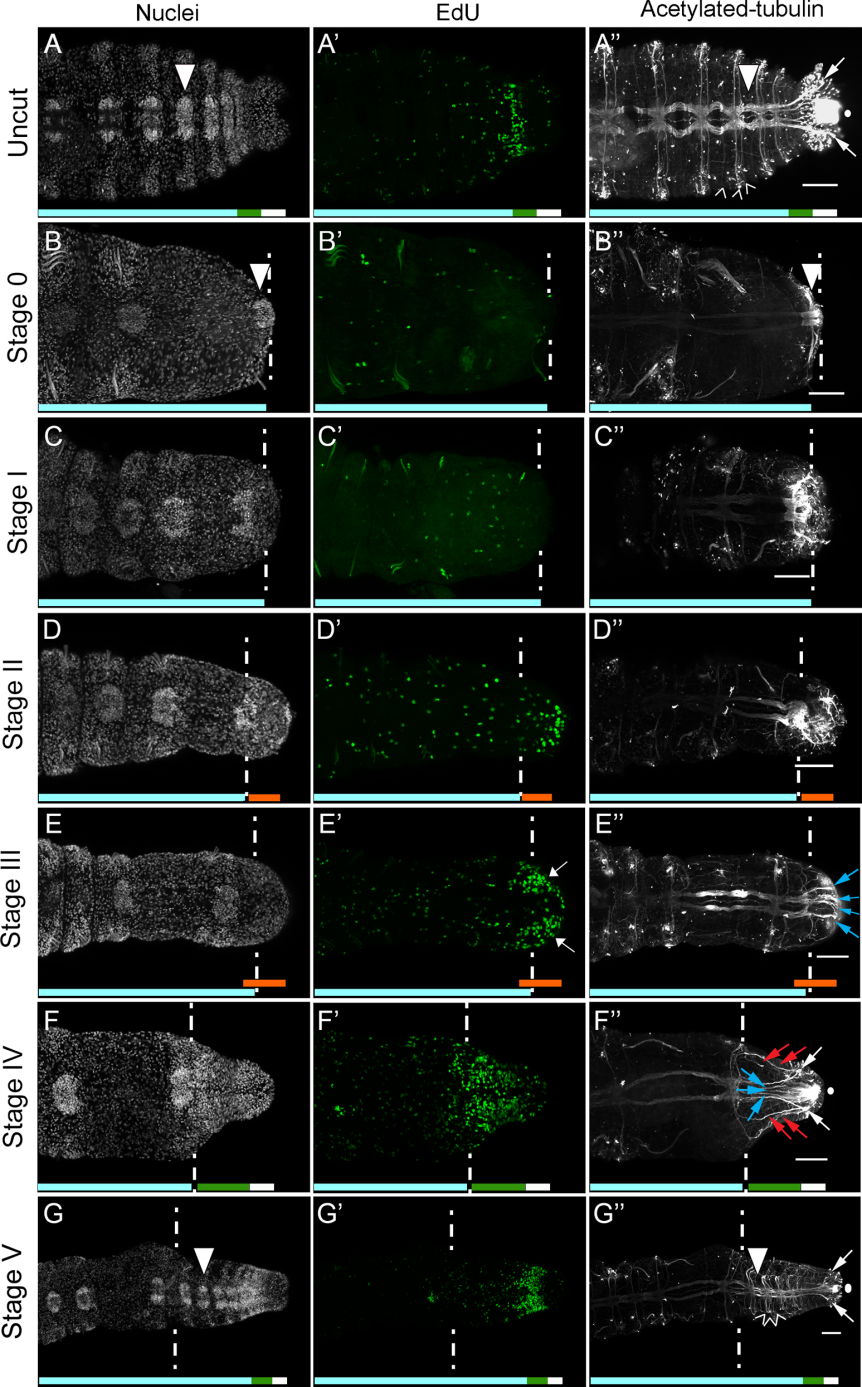
Blastema formation, cell division patterns and nerve cord dynamics during posterior regeneration of *C*. *teleta*. All panels show posterior ends of amputated juveniles in ventral view, with anterior to the left. Amputations were conducted at the boundary of segment 10 and 11. White dotted lines indicate approximate position of amputation, and all tissue to the right of dotted lines is regenerated tissue. The panels in each row are from a single individual. The regenerative process is described as progressing through different stages (left of rows), and specific stain, chemical or antibody is indicated at the top of columns. (A-G) Hoechst 33342 staining showing nuclei; (A’-G’) EdU incorporation marking dividing cells; (A”-G”) anti-acetylated α-tubulin labeling neurites. Scale bars in A”-G” are 50 μm. White circle in A”, F” and G” shows cilia of the hindgut. White arrowheads show mature ganglia in A, A”, B, B”, G and G”. Open arrowheads show peripheral nerves in A” and G”. White arrows in A”, F” and G” mark the Y-shaped neurites which extend into the pygidium. Blue arrows in E” mark nerve tracts. Blue arrows in F” mark medial axons that branch from the ventral nerve cord and red arrows mark axons that branch from lateral nerves. White arrows in E’ indicate bilateral clusters of EdU-positive cells. Chaetae are autofluorescent and are visible in A’, B’, C’ and D’.

The posterior end of unamputated juveniles contains mature abdominal segments (aqua bar in Fig 2A–2A”), a subterminal posterior growth zone (PGZ; green bar in Fig 2A–2A”), and a terminal pygidium (white bar in Fig 2A–2A”). The ventral nerve cord of each mature abdominal segment contains a ganglion (Fig 2A and 2A”, white arrowhead marks one in each panel), with three peripheral nerves extending laterally (Fig 2A”, open arrowheads showing three peripheral nerves). In uncut juveniles, we define the PGZ as the area anterior to the pygidium, but posterior to the posterior-most segment containing a well-defined ganglion. Segments immediately anterior to the PGZ tend to be smaller than segments further anterior, consistent with them undergoing significant growth after segment boundaries initially form. In the posterior end of the animal, the lumen of the hindgut contains cilia that can be visualized by anti-acetylated α-tubulin (Fig 2A”, white circle; S2A Fig). The pygidium of *C*. *teleta* is simple in morphology and lacks terminal projections such as anal cirri. Neurites extend from the posterior-most ganglion in a posterior direction into the pygidium. The lateral-most of these have a Y-shaped pattern as they extend through the PGZ, and innervate the pygidium with a diagonal orientation with respect to the anterior-posterior axis of the body (Fig 2A”, white arrows).

Transverse amputation at the boundary of segment 10 and 11 (Stage 0), removes 13–18 abdominal segments, the PGZ and pygidium (Fig 2B–2B”, aqua bar indicates the remaining segments; white arrowheads in B and B” mark the ganglion of segment 10). In addition, the ciliated hindgut is removed (S2B Fig), and the axons of the ventral nerve cord are abruptly terminated (Fig 2B”). In amputated animals, EdU-positive cells are scattered throughout the body, including in segments anterior to the amputation site, which is typical for actively growing two-week old juveniles (Fig 2B’). Stage I of regeneration (approximately 24 hours post-amputation (p.a.)), is characterized by several features. Wound healing is initiated within approximately 4–6 hours post-amputation, and occurs by contraction of the severed edges of the body wall. At Stage I, an epithelium covers the amputation site. In addition, the pattern of EdU-positive cells changes relative to uncut animals. EdU-positive cells are still scattered throughout the body (Fig 2C’), however there is a reduction in EdU-positive cells near the cut site. When the ratio of EdU-positive cells to total cells are quantified in the three segments proximal to the cut site (segment 8, 9 and 10), there is a significant decrease in the proportion of dividing cells compared to homologous segments in uncut controls (segment 8, *p =* 0.012; segment 9, *p* = 0.006; segment 10, *p* = 0.001; S1B Fig). Acetylated-tubulin positive neurites appear damaged by the amputation, visible by their disorganized appearance immediately anterior to the amputation site (Fig 2C”).

Stage II of regeneration begins at approximately 2 days p.a. At this stage, there is no longer a significant difference in the ratio of EdU-positive cells to total cells in the three segments closest to the wound site compared to uncut controls (segment 8, *p =* 0.288; segment 9, *p* = 0.165; segment 10, *p* = 0.172; S1B Fig). A small blastema containing numerous dividing cells is now visible immediately posterior to the amputation site (Fig 2D–2D”, orange bars). Coincident with the appearance of a blastema containing dividing cells, neurites are dispersed throughout the blastema (Fig 2D”). The morphology of the longitudinal nerves enable the location of the amputation site to clearly be seen; the longitudinal nerves are thicker immediately anterior to the amputation site, and there is an abrupt transition at the amputation site, with thinner nerves posterior to the amputation site (compare longitudinal nerves to the left and right of the dotted lines in Fig 2D”, 2E” and 2F”). This transition is clearly visible from Stage II–IV of regeneration (approximately 2–5 days p.a.). These appear to extend from severed axons of the ventral nerve cord, and form a random ‘web’ throughout the blastema. The hindgut appears very similar to the hindgut at a similar position in uncut animals (S2C Fig).

During Stage III of regeneration (approximately 3 days p.a.), there is more organization within the blastema, both in the distribution of proliferating cells and the pattern of axonal projections. Proliferating cells are densely packed within the nascent tissue, forming two bilateral clusters, which extend anterior of the boundary between the blastema and the pre-existing segmented tissue (Fig 2E’, white arrows; Fig 2E–2E”, orange bars). At Stage III, there is also a significant increase in the ratio of EdU-positive cells to total nuclei in the 10^th^ and 9^th^ segments (but not the 8^th^ segment), when compared to uncut controls (segment 8, *p* = 0.176; segment 9, *p* = 0.046; segment 10, *p* = 0.025; S1B Fig). Numerous axonal projections in the blastema are now organized into multiple axon tracts, oriented along the anterior-posterior axis, and each comprised of multiple neurites (Fig 2E”, blue arrows; compare Fig 2D” and 2E”). Some preparations were observed to have a few cilia in the endothelial lining of the lumen of the gut within the blastema at stage III, however most preparations did not (S2D Fig).

At Stage IV (approximately 5 days p.a.), the blastema contains numerous dividing cells, organized in bilateral clusters that extend anterior to the cut site, and are less dense at the posterior end of the nascent tissue (Fig 2F’). Stage IV is marked by a characteristic pattern of neuronal projections into the blastema. Approximately 4 lateral axon tracts extend from pre-existing lateral nerves (2 on each side; Fig 2F”, red arrows). There are five medial axon tracts that extend from the ventral nerve cord into the posterior end of the regenerating tissue (Fig 2F”, blue and white arrows). Notably the two lateral-most neurites extending from the ventral nerve cord form a Y-shaped pattern, reminiscent of the Y-shaped branching pattern seen at the posterior end of uncut animals (Fig 2F”, white arrows). In light of neurite morphology and the decreased density of EdU-positive cells at the posterior end, we postulate that Stage IV marks the beginning of the reformation of the PGZ and pygidium (Fig 2F–2F”, green and white bars, respectively). At this stage, a ciliated hindgut has also reformed (S2E Fig). Similar to Stage III, at Stage IV there is a significant increase in cell proliferation in the 9^th^ and 10^th^ segments, compared to corresponding segments in uncut controls. Unlike Stage III, at Stage IV this increase is also seen in the 8^th^ segment when compared to the corresponding segments in uncut controls (segment 8, *p* = 0.023; segment 9, *p* = 0.0049; segment 10, *p* = 0.00007; S1B Fig). The ratio of EdU-positive cells to total nuclei in the blastema, when comparing across Stages II-IV, are not significantly different from each other. EdU-positive cells are present in the ectoderm, mesoderm and endoderm of the blastema at all stages of regeneration.

Stage V of regeneration (approximately 7 days p.a.), is defined by the appearance of multiple new segments containing differentiated cell types (Fig 2G–2G”, aqua bar). New segments are visible by the presence of inter-segmental furrows (data not shown), ganglia (compare Fig 2C–2F with Fig 2G, white arrowhead marks one newly formed ganglia in Fig 2G and 2G”), and three pairs of peripheral axons extending laterally within each newly formed segment, which re-establishes the organization of peripheral nerves characteristic for abdominal segments (Fig 2G”, open arrowheads). Some preparations also contain newly formed chaetae in the segments closest to the wound site. The PGZ, pygidium and ciliated hindgut formed at Stage IV persists (Fig 2G–2G”, green and white bars, respectively). In the gut, cilia are present along the entire length of the regenerated gut tissue (S2F Fig). At Stage V, there is a significantly higher ratio of dividing cells in segments 9 and 10, but there is not a significantly different ratio of dividing cells in segment 8 compared to uncut controls (segment 8, *p* = 0.116; segment 9, *p* = 0.044; segment 10, *p* = 0.045; S1B Fig). It should be noted that the rate of regeneration varies somewhat among individuals, and preliminary data suggests it may be due, at least in part, to variation in nutrient intake. Therefore days post-amputation are approximate for each stage described.

### Expression of *Hox* genes in regenerating tissue following transverse amputation

**Fig 3:**
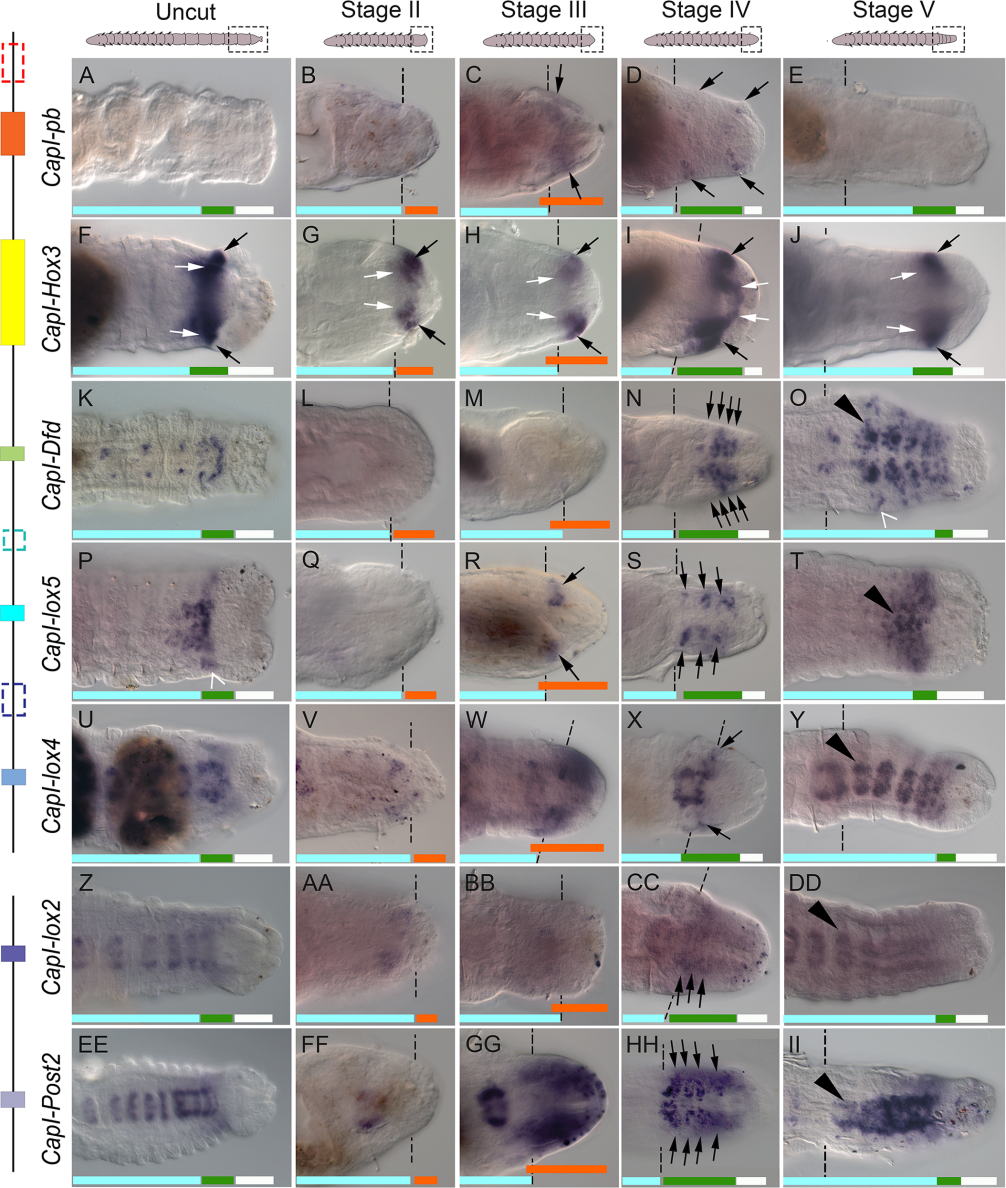
Most *Hox* genes are expressed in specific domains in the blastema. All panels show posterior ends of amputated juveniles in a ventral view, with anterior to the left. Amputations were conducted at the boundary of segment 10 and 11. Dotted lines indicate approximate position of amputation, and all tissue to the right of these lines is regenerated tissue. The amputation site in T and DD is not marked as it is outside the field of view. The stage of regeneration is indicated at the top of columns, and gene names are to the left of rows. Schematics of juveniles are shown at the top of columns, with dotted rectangles indicating approximate position of images shown. A schematic representation of the genomic organization is shown to the left of gene names. Black lines depict the two scaffolds containing *Hox* genes, and colored rectangles depict the coding sequences of each *Hox* gene. Rectangles with dotted lines denote genes that are not expressed in the blastema during regeneration (*CapI-lab*, *CapI-Scr* and *CapI-Antp*). (A-E), *CapI-pb* expression; (F-J), *CapI-Hox3*; (K-O), *CapI-Dfd;* (P-T), *CapI-lox5*; (U-Y), *CapI-lox4*; (Z-DD), *CapI-lox2*; (EE-II), *CapI-Post2*. Dark shapes in D, E, F, I, M, R, U and FF are mud deposits within the lumen of the gut. Panels C-E, F, K, P-S, V-Y, BB-DD and II were generated using Helicon focus to combine 2–5 focal planes. Aqua bars indicate mature segments, green bars indicate the posterior growth zone, orange bars indicate zones of proliferating cells, and white bars indicate the pygidium. Black arrowheads in O, T, Y, DD and II indicate mature ganglia. Black arrows in C-D, F-J, N, R, S, X, CC and HH indicate ectodermal expression, and white arrows in F-J indicate mesodermal expression. Open arrowhead in O indicates expression in individual ectodermal cells lateral to the newly formed ganglia, and open arrowhead in P indicates expression in ectodermal cells that extend laterally in a band on either side of the ventral midline in the PGZ.

To investigate the possible involvement of *Hox* genes in formation, patterning and outgrowth of the blastema, we analyzed the expression of each *Hox* gene following transverse amputation between segment 10 and 11 (Fig 3). Expression was examined at four stages of regeneration, as defined in the preceding Results section “Posterior regeneration in *C*. *teleta*”. The stages examined were Stage II (approximately 2 days p.a.), when a blastema with proliferating cells becomes morphologically visible (see Fig 2D and 2D’; Fig 3B, 3G, 3L, 3Q, 3V, 3AA and 3FF), Stages III and IV (approximately 3 days and 5 days p.a.), during outgrowth of the blastema but prior to the formation of nascent ganglia (see Fig 2E–2F”; Fig 3C and 3D, 3H and 3I, 3M and 3N, 3R and 3S, 3W and 3X, 3BB and 3CC and 3GG and 3HH), and Stage V (approximately 7 days p.a.), when new segments and ventral nerve cord ganglia have formed (see Fig 2G–2G” and Fig 3E, 3J, 3O, 3T, 3Y, 3DD and 3II). Seven *Hox* genes are expressed in the blastema during regeneration, and each displays a characteristic expression pattern. *CapI-lab*, *CapI-Scr* and *CapI-Antp* were not detectable in the blastema at the time points analyzed (data not shown; see dotted rectangles in Fig 3 and S3 Fig showing their relative positions within the *Hox* cluster). The timing in onset of gene expression in the blastema differs among *Hox* genes. *CapI-Hox3* initiates expression at Stage II, *CapI-pb*, *CapI-lox5*, *CapI-lox4* and *CapI-Post2* initiate expression at Stage III, and *CapI-Dfd* and *CapI-lox2* are expressed by Stage IV of regeneration. At Stage V, the expression of each of the genes generally resembles their expression pattern in uncut animals (with the exception of *CapI-Dfd*, as noted below; see S3 Fig). Although broadly speaking, while there are some similarities in *Hox* gene expression during blastemal outgrowth, each gene displays a unique and identifiable expression pattern within the regenerating tissue.

At Stage II of regeneration, only *CapI-Hox3* is detectable (Fig 3G; no expression of *CapI-Hox3* is detected at 1 day p.a., data not shown). At this stage, a blastema is morphologically visible, and contains multiple dividing cells and numerous disorganized neurites (see Fig 2D–2D”; orange bar in Fig 3B, 3G, 3L, 3Q, 3V, 3AA and 3FF indicates zone of proliferating cells). *CapI-Hox3* expression is initiated in ventral ectodermal cells and mesodermal cells in two bilateral clusters, spanning an area from posterior to the cut site (dotted lines) through approximately half of the blastema, leaving an area at the posterior-most end of the blastema free of *CapI-Hox3* expression (Fig 3G; black and white arrows indicate ectodermal and mesodermal populations, respectively).

At Stage III of regeneration, *CapI-pb*, *CapI-lox5*, *CapI-lox4* and *CapI-Post2* are expressed in ventral ectodermal cells of the blastema, and *CapI-Hox3* shows persistent expression in both ectodermal and mesodermal cells (Fig 3C, 3H, 3M, 3R, 3W, 3BB and 3GG; black and white arrows in Fig 3H denote ectodermal and mesodermal cell populations, respectively). At this stage, the blastema contains numerous proliferating cells and neuronal processes (Fig 2E–2E”; orange bar in Fig 3C, 3H, 3M, 3R, 3W, 3BB and 3GG indicates zone of proliferating cells). *CapI-pb* expression is somewhat variable between samples. In a subset of cases (n = 16/25), it is in ectodermal cells that form two diffuse bilateral clusters immediately posterior to the wound site (Fig 3C; black arrows), and in the other cases it is not detectable (data not shown). *CapI-Hox3* is expressed in a broader domain than *CapI-pb*, in bilateral clusters in both ectodermal and mesodermal cell populations (Fig 3H, black and white arrows, respectively). As in Stage II, the *CapI-Hox3* expression domain is localized close to the cut site, with the posterior end of the blastema free of expression. *CapI-lox5* is expressed in two small bilateral clusters in the ventral ectoderm of the blastema, posterior to the cut site (Fig 3R, black arrows). The expression pattern of *CapI-lox5* broadly resembles that of *CapI-pb*, although *CapI-lox5* expressing cells form more defined clusters than those that express *CapI-pb* (compare Fig 3C and 3R). *CapI-lox4* is expressed in a diffuse domain in the ventral ectoderm (Fig 3W), and covers a larger area of the blastema compared with the expression domain of *CapI-pb* (Fig 3W; compare Fig 3C and 3W). *CapI-Post2* is expressed in ectodermal tissue, extending from the cut site to the posterior-most end of the blastema in two bilateral clusters. The medial boundary of these two clusters is close to the ventral midline but there is a small gap in expression between the two domains (Fig 3GG).

*Hox* All seven genes described are expressed in the blastema at Stage IV. At Stage IV, the blastema has increased in size, and a PGZ and pygidium have formed, although no mature segments are visible (Fig 2F–2F”; green and white bars in Fig 3D, 3I, 3N, 3S, 3X, 3CC and 3HH indicate the PGZ and pygidium, respectively). *CapI-pb* is expressed in a small subset of cases (n = 4/21), in two bands of ectodermal cells; one immediately adjacent to the wound site, and a second one immediately anterior to the posterior end (Fig 3D, black arrows; the remaining cases do not show blastemal expression). As in Stage III, *CapI-Hox3* is expressed in both ectodermal and mesodermal cell populations. Expression is approximately halfway between the wound site, and the posterior end of the blastema (Fig 3I, black and white arrows indicate ectodermal and mesodermal cell populations, respectively). *CapI-Dfd* appears for the first time in the blastema at Stage IV in approximately half the animals assayed (n = 7/13). In these animals, *CapI-Dfd* is expressed in groups of ectodermal cells that radiate from the midline in approximately four transverse rows on each side (Fig 3N, black arrows). *CapI-lox5* is evident in approximately three bilateral cell clusters (Fig 3S, black arrows), beginning immediately adjacent to the cut site, and extending approximately three quarters of the length of the blastema along the anterior-posterior axis. This pattern broadly resembles the repeated pattern of *CapI-Dfd* expression, although there are some differences. Adjacent *CapI-lox5* expressing cell clusters are situated further apart from each other than the transverse bands of *CapI-Dfd* expression, and there are fewer bands of *CapI-lox5* expressing cells (compare Fig 3N and 3S). *CapI-lox4* is detected in a single, narrow band of ectodermal cells with a medial-lateral orientation, at the boundary of the cut site (Fig 3X, black arrows). *CapI-lox2* expression is initiated at this stage, in multiple medial-lateral bands of ectodermal cells in the blastema (Fig 3CC, black arrows). Compared to its single broad expression domain at Stage III, *CapI-Post2* is now expressed in transverse bands, with expression domains that extend ventro-laterally, but do not meet at the midline (Fig 3HH, black arrows).

At Stage V, several new segments have formed in the regenerated tissue (Fig 2G–2G”; Fig 3E, 3J, 3O, 3T, 3Y, 3DD and 3II, aqua, green and white bars indicate mature segments, PGZ and pygidium, respectively). Six *Hox* genes are expressed in the regenerated tissue at this stage, and *CapI-pb* is no longer detectable in the regenerated tissue of any animal (Fig 3E). *CapI-Hox3* is expressed within the PGZ, in both the ectoderm and mesoderm, with no expression visible in the pygidium or in the nascent segments (Fig 3J, green bar indicates the PGZ). *CapI-Dfd* and *CapI-lox5*, are also expressed in the newly formed PGZ (Fig 3O and 3T; green bars denote the PGZ). *CapI-Dfd*, *CapI-lox5*, *CapI-lox4*, *CapI-lox2* and *CapI-Post2* are each expressed in the newly formed ganglia (*CapI-Dfd*, Fig 3O; *CapI-lox5*, Fig 3T; *CapI-lox4*, Fig 3Y; *CapI-lox2*, Fig 3DD; *CapI-Post2*, Fig 3II; black arrowheads show an example in each panel, aqua bars denote mature segments).

Of the ten *Hox* genes surveyed, seven are expressed in regenerating tissue, and three of these seven are also expressed in the PGZ of growing animals. *CapI-Dfd* and *CapI-lox5* are expressed in ectodermal cells of the PGZ in uncut animals and ectodermal cells of the blastema during regeneration (Fig 3K and 3P; see aqua, green and white bars denoting mature segments, posterior growth zone, and pygidium, respectively), and *CapI-Hox3* is expressed in both ectodermal and mesodermal cells of the PGZ, and also in ectodermal and mesodermal cells in the blastema (Fig 3F). While most genes show a similar pattern between the posterior portion of uncut animals and Stage V of regenerates (for example, compare Fig 3A and 3E and Fig 3EE and 3II), the expression of *CapI-Dfd* differs. At Stage V, *CapI-Dfd* is expressed in a broader domain along the medial-lateral axis than that seen in uncut animals (compare Fig 3K and 3O), and is in individual cells in a lateral position (Fig 3O, open arrowhead shows an example). Our results show that most *Hox* genes are expressed in the blastema following transverse amputation, with different spatial and temporal characteristics. By Stage V of regeneration, the expression pattern of the *Hox* genes in regenerating tissue has returned to that of uncut animals.

### Stable expression of *Hox* genes in pre-existing segments following amputation at the segment 10/11 boundary

**Fig 4:**
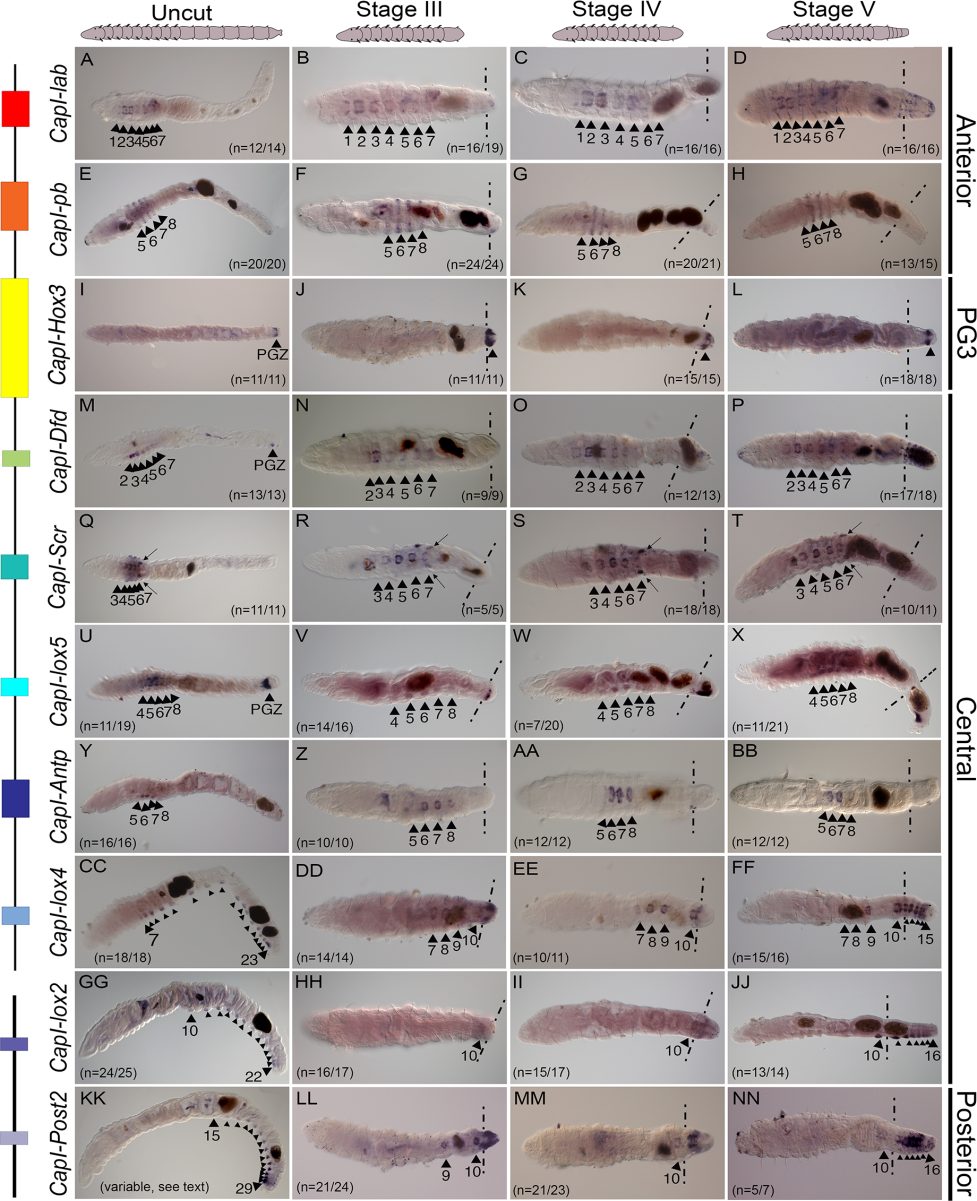
*Hox* gene expression domains remain stable after transverse amputation, with the exception of *CapI-Post2*. Panels E-H, M, V-X, Y, CC, GG, JJ and KK, are lateral views with anterior to the left, and all other panels are ventral views with anterior to the left. Where appropriate, approximate amputation sites are marked with dotted lines, and all tissue to the right of these lines is newly formed tissue. The stage of regeneration is shown at the top of columns, and the gene name to the left of rows. Schematics at the top of columns represent the images shown in panels. To the right of panels, the class to which each *Hox* gene belongs is denoted. A schematic representation of the genomic organization is shown to the left of gene names. Black lines depict the two scaffolds containing *Hox* genes, and colored rectangles depict the coding sequences of each *Hox* gene. Black arrowheads with associated numbers indicate the segment number in which expression is seen. The number of cases representing the expression pattern shown are noted at the bottom right hand corner, or bottom left hand corner of panels. Arrows in Q-T indicate expression in the paired genital ducts. Dark brown shapes within juveniles are deposits within the lumen of the gut. PGZ denotes expression in the posterior growth zone in panels I, M and U. In the panels showing *CapI-lox5* expression, the remainder of cases not representing the expression pattern shown, were those where high levels of background in other tissues prevented expression in the ganglia from being clearly observed.

To determine the effect of amputation on the expression of *Hox* genes in pre-existing segments, we studied juveniles at 2 weeks post-metamorphosis (approximately 22–28 segments) and conducted cuts between the boundary of segments 10 and 11. This position is posterior to the first abdominal segment. Gene expression was examined at Stage III, IV and V of regeneration (approximately 3 days, 5 days and 7 days post-amputation), and compared to age-matched uncut controls. We observed stability of *Hox* gene expression boundaries in uncut juveniles irrespective of length, and even following amputation. A single exception is *CapI-Post2*, which shifted its anterior expression boundary in pre-existing segments following amputation (Fig 4).

**Fig 5:**
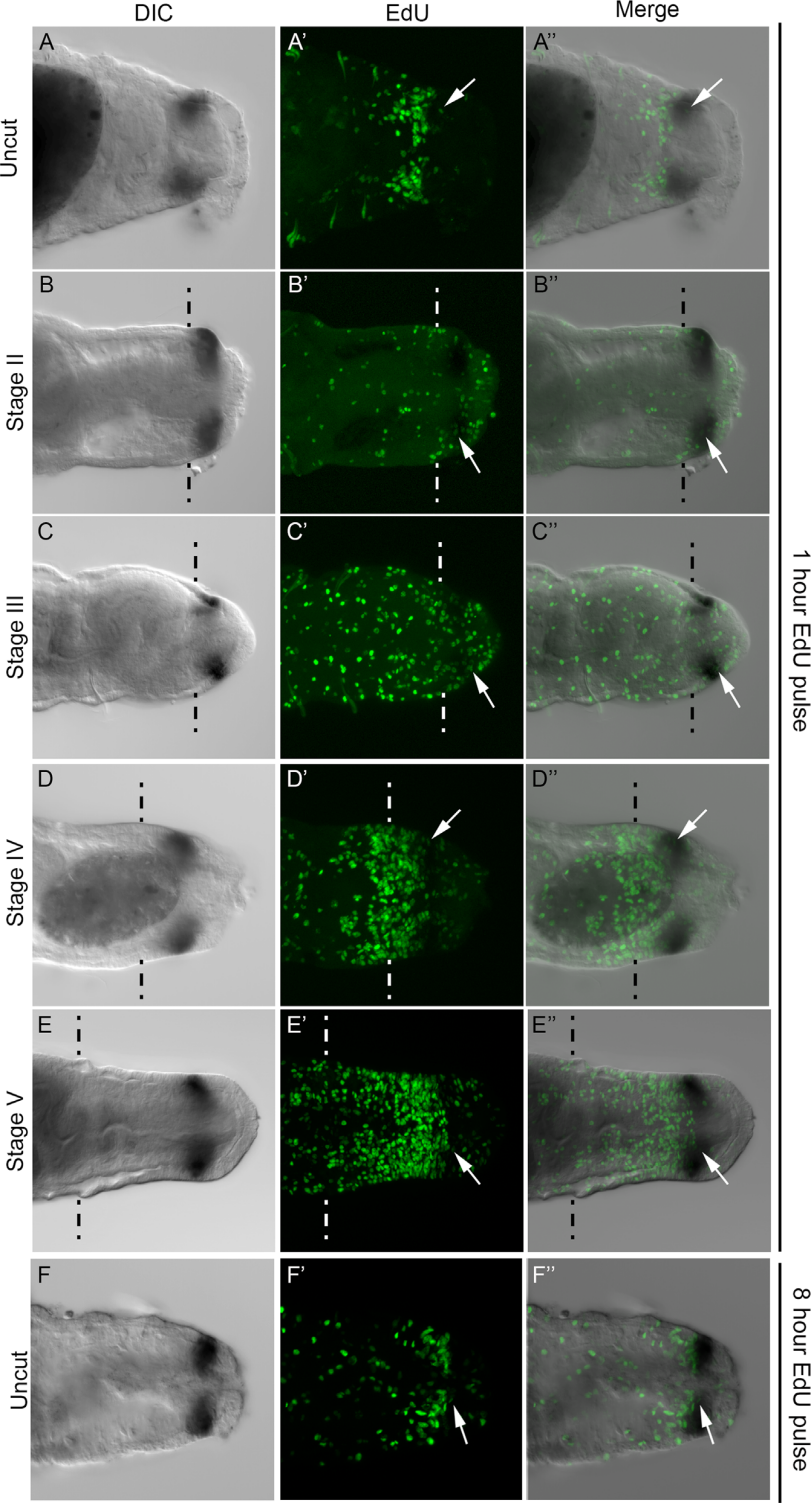
Relationship between *CapI-Hox3* and dividing cells in the blastema and PGZ. All panels show posterior ends of juveniles in a ventral view, with anterior to the left. Where appropriate, approximate amputation sites are marked with dotted lines, and all tissue to the right of these lines is newly generated tissue. All panels are confocal images. Images shown in panels A-F were generated from a subset of slices from a z-stack, whereas panels A’-F’ were generated from maximum projections of z-stacks. A DIC image of the *CapI-Hox3* expression domain is shown in the first column, EdU-positive cells in the second column, and a DIC/EdU merge shown in the final column. Stages of regeneration are indicated to the left of rows. (A-F) DIC image of *CapI-Hox3* expression; (A’-F’) EdU incorporation marking dividing cells; (A”-F”) Merge of DIC and EdU channels. (A-A”) Unamputated juveniles following a one hour EdU pulse; (B-B”) Stage II of regeneration; (C-C”) Stage III; (D-D”) Stage IV; (E-E”) Stage V; (F-F”) Unamputated juveniles following an eight hour EdU pulse. The panels in each row are from a single juvenile. Dark brown shapes within juveniles are deposits within the lumen of the gut. White arrows indicate examples of single EdU-positive cells within the *CapI-Hox3* expression domain.

In uncut 2 week post-metamorphic juveniles, the anterior class *Hox* genes, *CapI-lab* and *CapI-pb*, are expressed in thoracic segments 1–7 and 5–8, respectively, and *CapI-Hox3* (paralogy group 3 (PG3)), is expressed exclusively in the PGZ. *CapI-lab* expression is limited to the ganglia of the VNC, whilst *CapI-pb* expression is also apparent in the epidermis, in a striped pattern (Fig 4A, 4E and 4I). Although the anterior and posterior boundaries of these genes are invariant, *CapI-lab* expression is strongest in segments 2 and 3, and weaker in the surrounding segments, while *CapI-pb* shows strongest expression in segments 6 and 7, with weaker expression in segments 5 and 8. Following amputation, the anterior and posterior boundaries of expression of each gene were unchanged (Fig 4B–4D and Fig 4F–4H). In the case for *CapI-Hox3*, amputation removes the original expression domain, and *CapI-Hox3* expression was not observed in pre-existing segments during any time point examined. *CapI-Hox3* expression is initiated in the newly formed tissue at Stages III-V of regeneration (Fig 4J–4L; see also Fig 3G–3J and Fig 5).

The six *C*. *teleta* central class *Hox* genes, *CapI-Dfd*, *CapI-Scr*, *CapI-lox5*, *CapI-Antp*, *CapI-lox4* and *CapI-lox2*, are expressed in staggered domains in uncut animals, and together their expression encompasses almost the entire anterior-posterior body axis (see Fig 1B). In intact animals, most exhibit expression restricted to the ganglia of the VNC and growth zone. *CapI-Dfd* is expressed in the ganglia of segments 2–7 and the PGZ (Fig 4M). Although the anterior and posterior boundaries of expression are invariant, relative expression levels between segments differ for *CapI-Dfd;* expression is strongest in segments 3 and 4, but weaker in segments 2 and 5, 6 and 7. *CapI-Scr* is expressed in the ganglia of segments 3–7 with expression strongest in segments 5 and 6, but weaker segments 3, 4 and 7 (Fig 4Q). In addition, *CapI-Scr* is also expressed in the paired genital ducts located between segments 7 and 8, which are male reproductive structures ([15]; Fig 4Q, black arrows). *CapI-lox5* is expressed in the ganglia of segments 4–8 and the PGZ. Expression is strongest in segments 5 and 6, and weaker in segments 4, 7 and 8 (Fig 4U). *CapI-Antp* is expressed in the ganglia of segments 5–8, and expression appears greatest in segments 6 and 7 (Fig 4Y). *CapI-lox4* and *CapI-lox2* have different anterior expression boundaries, otherwise they both are expressed in the ganglia of all abdominal segments, regardless of the length of the animal. *CapI-lox4* has an anterior boundary in segment 7, and *CapI-lox2* has an anterior boundary in segment 10. The expression of *CapI-lox4* appears strongest in segments 7–9, and uniformly weaker in all abdominal segments, while the expression of *CapI-lox2* appears uniform across all segments (Fig 4CC and 4GG). Following amputation between segments 10 and 11, the expression boundaries of each central class *Hox* genes do not change, even though amputation disrupts their normal expression domain (Fig 4N–4P, 4R–4T, 4V–4X, 4Z, 4AA and 4BB, 4DD–4FF, 4HH–4JJ). For example, although the expression domain of *CapI-lox4* extends from segment 7 to the posterior end, amputation at the 10^th^ segment does not change the *CapI-lox4* anterior boundary, and expression remains in the ganglia of the pre-existing segments 7–10 at Stages III-V (Fig 4DD and 4EE). At Stage V, when new abdominal segments have formed, expression is also seen in all of the new ganglia (Fig 4FF).

Unlike each of the other *Hox* genes, which have stable anterior expression boundaries, the anterior expression boundary of *CapI-Post2* is variable in growing worms, and we observe a transient anterior shift in pre-existing tissue following amputation between segments 10 and 11. The posterior *Hox* gene *CapI-Post2* is consistently expressed in multiple abdominal segments and always includes the posterior-most segments. Anterior segments show weaker expression than posterior segments. A total of 55 uncut juveniles between 14 and 42 segments in length were examined for *CapI-Post2* expression, and the anterior boundary was seen to range from segment 10 (the first abdominal segment) to segment 22 (the 13^th^ abdominal segment) (Fig 4KK shows an example with an anterior expression boundary at segment 15; see also S5 Fig). Therefore, the anterior expression boundary is unrelated to the total length of the animal. When amputated at the boundary of segments 10 and 11, the anterior boundary of *CapI-Post2* expression shifts to include thoracic segment 9 by Stage III, and the posterior boundary of expression remains in the posterior-most segment (segment 10) (Fig 4LL). This expression represents a shift anterior by one segment relative to any expression seen in normally growing animals. By Stage IV, the anterior boundary of expression has shifted to the first abdominal segment, segment 10 (Fig 4MM). Similarly at Stage V, the *CapI-Post2* anterior boundary is in segment 10, and is also expressed in all newly formed abdominal ganglia (Fig 4NN).

### *CapI-Hox3* is expressed in a subset of dividing cells

*CapI-Hox3* expression is distinct from the spatial-temporal expression patterns of the other *Hox* genes, and is localized to the PGZ but is absent from the pygidium (Fig 5A). We conducted EdU incorporation experiments in combination with *in situ* hybridization to determine the relationship between the *CapI-Hox3* expression domain and dividing cells in the PGZ of uncut animals, and in the blastema during regeneration. For experiments involving regenerating animals, transverse amputations were conducted at the boundary of segment 10 and 11, and anterior pieces were collected at Stage II, III, IV and V of regeneration. Following collection of animals at each time point, a one hour pulse of EdU was administered, followed by fixation and *in situ* hybridization using *CapI-Hox3* as probe. For experiments involving uncut animals, either a one hour pulse or an eight hour pulse of EdU was administered, followed by fixation and *in situ* hybridization using *CapI-Hox3* as probe.

Following a one hour pulse of EdU, the relationship between the position of the *CapI-Hox3* expression domain and that of dividing cells was analyzed. The *CapI-Hox3* expression domain is immediately posterior to an area with a high-density of proliferating cells, and resides in an area with fewer dividing cells directly anterior to the pygidium (Fig 5A’ and 5A”). There are a few EdU-positive cells within the *CapI-Hox3* domain (Fig 5A’ and 5A”; white arrows show a single example). Similarly, after an eight hour pulse of EdU, the *CapI-Hox3* expression domain is in an area with few dividing cells compared with the zone directly anterior to it (Fig 5F–5F”). At Stage II of regeneration, when a small blastema becomes apparent, the anterior boundary of *CapI-Hox3* is adjacent to the cut site, and expression is absent from the posterior end of the nascent tissue (Fig 5B–5B”). Numerous EdU-positive cells are distributed throughout the blastema, including some within the *CapI-Hox3* expression domain (Fig 5B’ and 5B”; white arrows show a single example). Similarly, at Stage III of regeneration, *CapI-Hox3* is expressed in the anterior portion of the blastema within the field of EdU-positive cells, which are distributed throughout the blastema (Fig 5C–5C”, white arrows in Fig 5C’ and 5C” indicate an example of an EdU-positive cell within the *CapI-Hox3* expression domain). The pattern of EdU-positive cells has a non-uniform distribution within the nascent tissue by Stage IV; there is a low density of dividing cells at the posterior-most end of the nascent tissue and a high density of EdU-positive cells close to the cut site (Fig 5D’; see also Fig 2F–2F”). *CapI-Hox3* is expressed approximately halfway between the cut site and the posterior end of the blastema, within the area containing a low density of dividing cells. Its anterior boundary abuts the posterior edge of the domain of high density EdU-positive cells. There are some EdU-positive cells visible within the *CapI-Hox3* expression domain (Fig 5D–5D”; white arrows in D’ and D”). At Stage V of regeneration when several new segments have formed (see Fig 2G–2G”), *CapI-Hox3* is expressed in a pattern resembling that in uncut animals. Expression is directly anterior to the pygidium, and demarcates a subdomain within the PGZ that has a low density of dividing cells (Fig 5E–5E”). At this stage there are also some EdU-positive cells within the *CapI-Hox3* expression domain (white arrows in Fig 5E’ and 5E”).

### Anterior shift of *Hox* gene expression boundaries following transverse amputation

**Fig 6:**
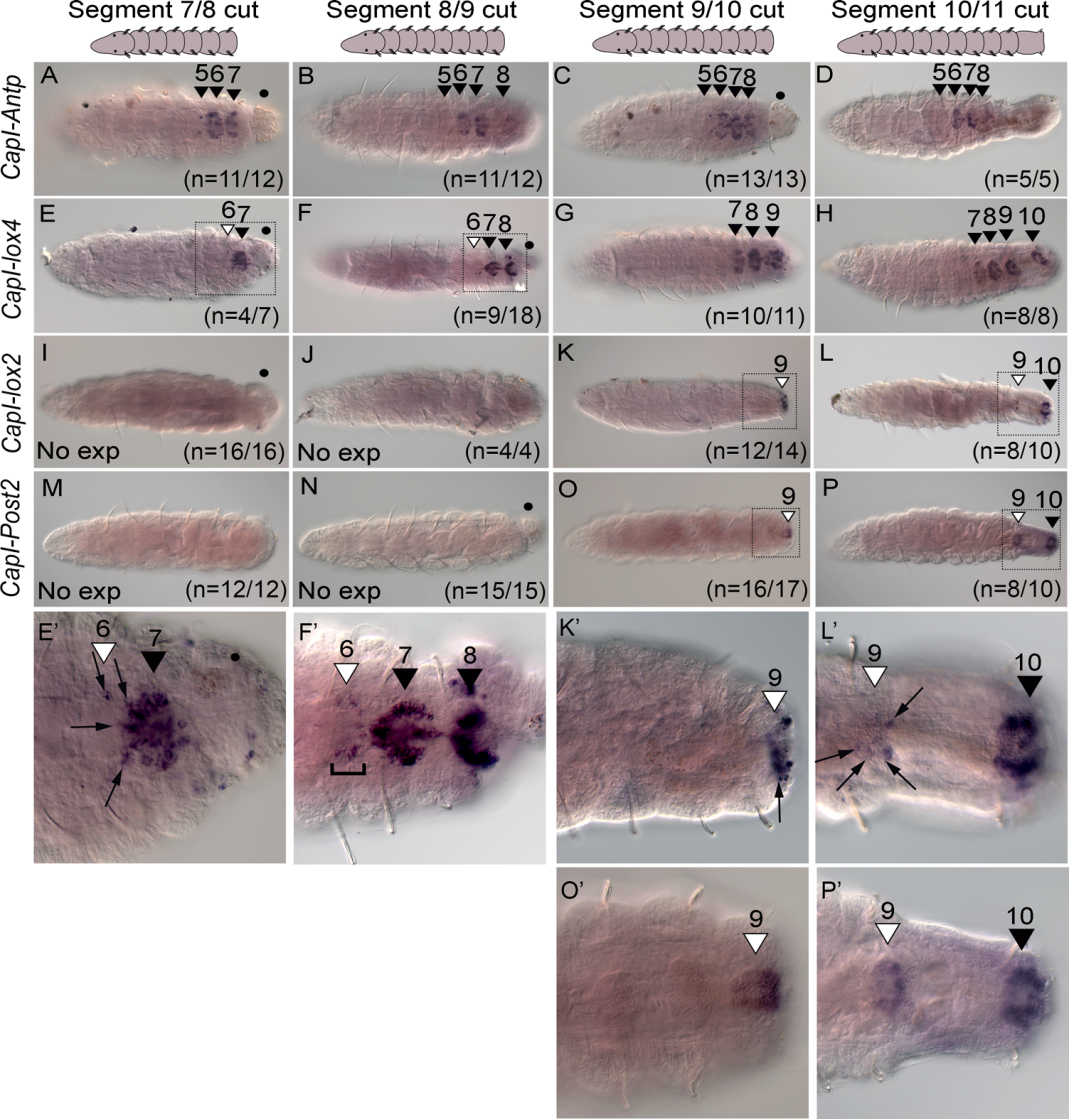
Amputation at different axial positions changes the anterior expression boundary of *CapI-lox4*, *CapI-lox2*, and *CapI-Post2* by 24 hours post-amputation. All panels show a ventral view, with anterior to the left. The position of amputation is indicated at the top of columns, and gene names to the left of rows. Schematics at the top of columns represent the images shown. (A-D) *CapI-Antp* expression; (E-H) *CapI-lox4*; (I-L) *CapI-lox2* and (M-P) *CapI-Post2*. Black arrowheads with associated numbers indicate the segment number in which ganglionic expression is seen in uncut animals, while white arrowheads with associated numbers indicate the segment number in which an anterior shift in ganglionic expression is seen. If no expression is seen, this is indicated in the bottom right corner of panels (I, J, M and N; No exp). The number of cases representing the expression pattern shown are noted at the bottom right hand corner of panels. Panels E’, F’, K’, L’, O’ and P’ are high magnification images of posterior ends of juveniles indicated by dotted rectangles in panels E, F, K, L, O and P, respectively. Gut protruding from the site of amputation is indicated by a black dot (•). Panels B and D were created using Helicon focus (3 and 4 focal planes respectively).

**Fig 7:**
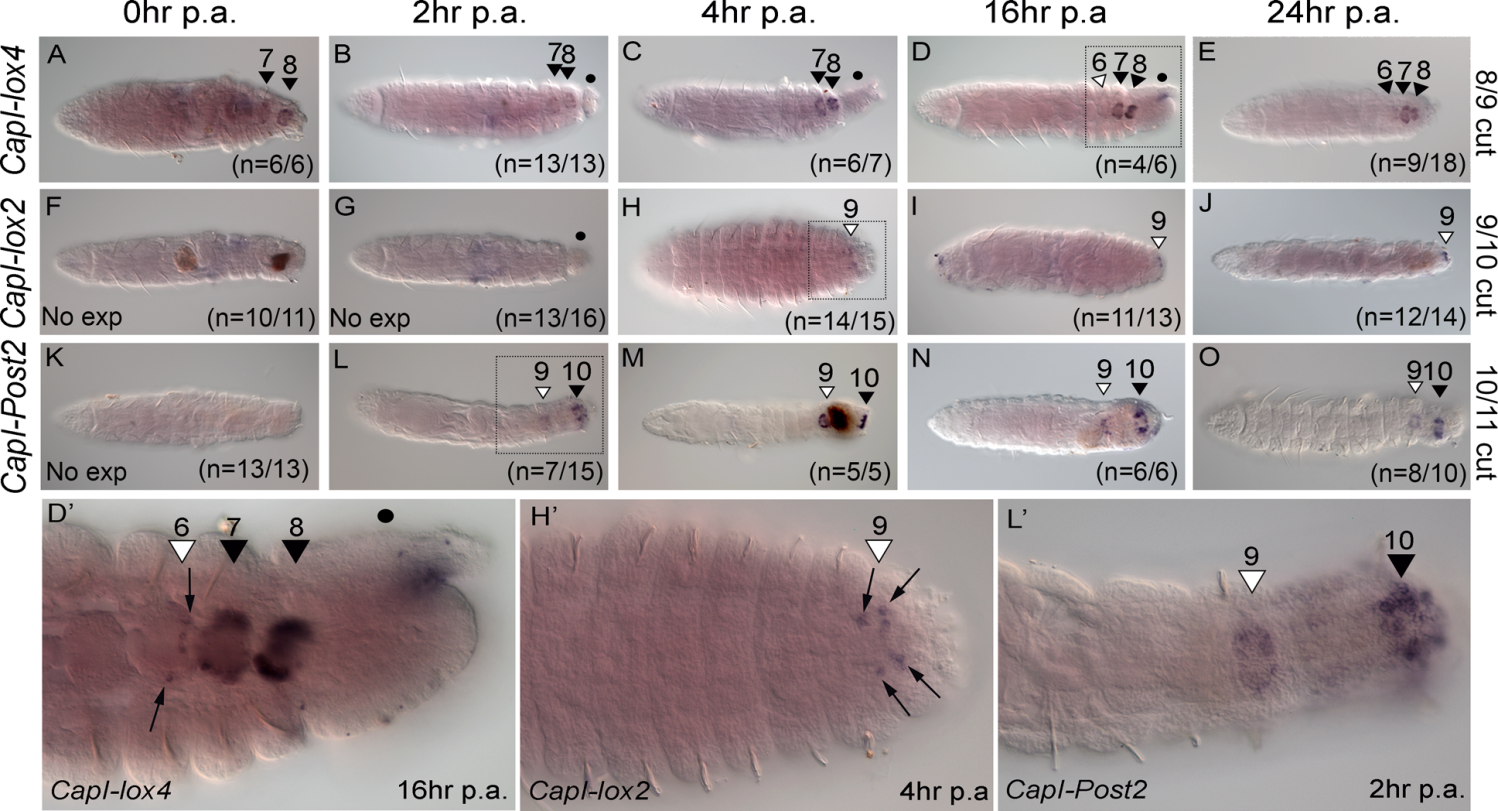
Timing of anterior shift in *CapI-lox2*, *CapI-lox4* and *CapI-Post2* expression boundaries is gene-specific. All panels show a ventral view, with anterior to the left. Amputations were conducted at the boundary of segment 8/9 (*CapI-lox4*), segment 9/10 (*CapI-lox2*) or segment 10/11 (*CapI-Post2*). The number of hours post-amputation (p.a.) is indicated at the top of columns, gene names to the left of rows and amputation site to the right of rows. (A-E) *CapI-lox4* expression; (F-J) *CapI-lox2*; (K-O) *CapI-Post2*. Panels D’, H’ and L’ are high magnification images of posterior ends of juveniles indicated by dotted rectangles in panels D, H and L respectively. In D’, H’ and L’, hours post-amputation is indicated in the bottom right corner, and gene name in the bottom left corner. Black arrowheads and associated numbers indicate the segment number in which ganglionic expression is seen in uncut animals, while white arrowheads indicate the segment number where an anterior shift in ganglionic expression is seen. No expression is indicated by ‘No exp’ in the bottom left corner of panels F, G and K. The number of cases supporting the expression patterns presented are shown in the bottom right hand corner of panels. Gut protruding from the site of amputation is indicated by a black dot (•) in B, C, D, G and D’. Dark shapes in F, M and N are mud deposits within the lumen of the gut. Panels F, G and L were created with Helicon focus from 2 focal planes.

To determine if the axial position of amputation has any effect on *Hox* gene expression in pre-existing segments, we conducted cuts at various positions along the anterior-posterior axis, and assayed expression of *Hox* genes at 24 hours p.a. (Fig 6 and S4 Fig). This time point was chosen since we observed the anterior shift in *CapI-Post2* within 2 hours following amputation (see below and Fig 7L), and we reasoned that a shift in expression of other *Hox* genes would be apparent by 24 hours. Most *Hox* genes do not show a shift in expression boundary following cuts at the boundary between 7 and 8, 8 and 9, 9 and 10 or 10 and 11 at 24 hours post-amputation (data not shown and Fig 6). For example *CapI-Antp* is expressed in the ganglia of segments 5 to 8 (Fig 6A–6D; black arrowheads), following amputation at any of these positions, the same pattern as observed in uncut juveniles. This result is similar to amputations conducted at the boundary of segments 10 and 11 (Fig 4).

However, three genes, *CapI-lox4*, *CapI-lox2* and *CapI-Post2*, show a shift in their anterior boundary of expression, and this shift depends upon the axial position of amputation. In unamputated juveniles, *CapI-lox4* is expressed in the ganglia of segment 7 to the posterior end of the animal (see Fig 1B and Fig 4CC). However, when juveniles are amputated between segments 7 and 8 or 8 and 9, the anterior expression boundary of *CapI-lox4* is now the ganglia of segment 6, in approximately half the cases (n = 4/7 and n = 9/18 respectively; the other cases show an anterior boundary at segment 7), and extends to the posterior end of the animal (segment 8 or 9; Fig 6E, 6E’, 6F and 6F’). When the amputation is conducted at the boundary of segment 9 and 10, or segment 10 and 11, an anterior shift in expression does not occur, and *CapI-lox4* is expressed from segment 7 to the posterior-most ganglia in almost all individuals (n = 10/11 and 8/8 respectively; one case shows an anterior boundary at segment 6; Fig 6G and 6H). In unamputated juveniles, *CapI-lox2* is expressed in the ganglion of segment 10, and in posterior abdominal segments (see Fig 1B and Fig 4GG). If juveniles are amputated between either segment 7 and 8, or 8 and 9, and analyzed at 24 hours post-amputation, no expression of *CapI-lox2* is detected. This is expected as the original expression domain has been removed (Fig 6I and 6J). However, if amputation is at the boundary of segment 9 and 10, or 10 and 11, the anterior boundary of expression shifts to include the ganglion of segment 9 in almost all individuals examined (n = 12/14 and n = 8/10 respectively. The remaining cases show either no expression (n = 2/14) or expression in segment 10 only (n = 2/10); Fig 6K, 6K’, 6L and 6L’). For amputations conducted at the boundary of segment 10 and 11, this anterior shift in expression is no longer apparent at 2 days p.a. (data not shown).

The anterior boundary of *CapI-Post2* expression in uncut juveniles is variable, but does not extend anterior to the ganglion of segment 10 (see S5 Fig). As expected, if transverse amputation is conducted between either segment 7 and 8, or 8 and 9, no expression of *CapI-Post2* is detectable, due to the original expression domain being removed (Fig 6M and 6N). However, as shown in Fig 4LL–4NN, if amputation is conducted at the boundary of segment 10 and 11, the anterior expression boundary shifts to include segment 9 in the majority of cases, and this shift occurs within 24 hours post-amputation (Fig 6P and 6P’; n = 8/10; two cases show expression in segment 10 only). In addition, amputation between segments 9 and 10 induces a shift in the anterior boundary of expression, so that *CapI-Post2* is expressed in the ganglion of segment 9 (n = 16/17; 1 case shows no expression; Fig 6O and 6O’).

### Timing of the shift in anterior boundary of expression of *CapI-lox4*, *CapI-lox2* and *CapI-Post2*

Since amputation at specific axial positions induced a new anterior boundary of expression for *CapI-lox4*, *CapI-lox2* and *CapI-Post2*, we sampled animals at several time points following amputation, to determine when this shift occurs. Five time points within 24 hours were examined following amputation (Fig 7). When amputated between segments 8 and 9, expression of *CapI-lox4* is seen in the ganglia of segment 7 onwards for the first 4 hours following amputation (Fig 7A–7C), similar to what is seen in unamputated juveniles of similar age (Fig 4CC). However, at 16 hours post-amputation, ectopic expression in segment 6 is first detected (n = 4/6; 2 cases show expression in segment 7 and 8 only; Fig 7D) and it persists until at least 24 hours p.a. (Fig 7E; n = 9/18; 9 cases show expression in segment 7 and 8 only). A higher magnification image of this expression shows that approximately 6 cells in the posterior side of ganglia 6 express *CapI-lox4* (Fig 7D’, black arrows). The pattern of expression is identical at both 16 and 24 hours p.a.

We amputated juveniles at the boundary of segment 9 and 10, and analyzed onset of expression of *CapI-lox2* in the ganglion of segment 9. The original expression domain was removed and no expression is observed within the first 2 hours of amputation (Fig 7F and 7G). However, at 4 hours p.a., expression of *CapI-lox2* in segment 9 is observed (n = 14/15; one case showed expression in segment 8 and 9; Fig 7H and 7H’), and it persists until at least 24 hours p.a. (Fig 7I and 7J; 16 hours, n = 11/13, two cases showed no expression; 24 hours, n = 12/14, two cases showed no expression). In segment 9, *CapI-lox2* is expressed in multiple cells at the midline and the posterior portion of the ganglion (Fig 7H’), and this expression pattern is similar at 4 hours, 16 hours and 24 hours p.a.

Immediately following amputation at the boundary of segment 10 and 11, no *CapI-Post2* expression is detectable (Fig 7K). However, by 2 hours p.a., there is expression of *CapI-Post2* in the ganglion of segments 9 and 10 (Fig 7L; n = 7/15, seven cases showed expression in segment 10 only, one case showed no expression). This expression pattern is present at 24 hours p.a. (Fig 7M–7O; at 24 hours two cases showed expression in segment 10 only), and persists until 3 days p.a. (Fig 4LL). Expression in the ganglion of segment 9 extends from the anterior to the posterior boundary of the ganglion, and is similar to that in segment 10 across all time points analyzed.

## Discussion

We present a comprehensive study of *Hox* gene expression during regeneration in the polychaete annelid, *C*. *teleta*, and interpret these expression data in the context of descriptions of cell proliferation and nervous system dynamics during the regenerative process. We show that *C*. *teleta* regenerates via cell proliferation and blastema formation (i.e. epimorphosis), a process concomitant with expression of a subset of *Hox* genes in the regenerating tissue. A blastema with proliferating cells is formed by Stage II (approximately 2 days p.a.), and new segments are formed by Stage V (approximately 7 days p.a.). Quantification of proliferating cells in the three segments closest to the wound site show that levels initially fall (by Stage I), recover to levels comparable to controls (by Stage II), and then rise over subsequent days (up to Stage V). From Stage 0-IV (the first 5 days of regeneration), neurites extend from pre-existing segments into the blastema, always maintaining contact with the posterior end of the new tissue. New ganglia appear by Stage V. During growth of the blastema, we observe unique expression of most of the *Hox* genes, suggesting a role in regeneration and patterning of new tissue. We also show that there is general stability of *Hox* expression domains in pre-existing segments following amputation, and that only three out of ten *Hox* genes shift their anterior boundaries of expression in the ventral nerve cord of pre-existing segments. This shift only occurs at some amputation positions, and is suggestive of limited morphallaxis.

### Cell proliferation and blastema formation during posterior regeneration of *Capitella teleta*

We investigated cell proliferation and blastema formation during posterior regeneration of *C*. *teleta*, using EdU incorporation as a marker of dividing cells. A morphologically distinguishable blastema appears by Stage II, and it is populated by numerous proliferating cells during all stages of outgrowth. This demonstrates that *C*. *teleta* undergoes posterior regeneration by epimorphosis. This is not a surprising finding, since the regeneration of most annelids involves localized cell division in the blastema [6,22–25]. Cells involved in regeneration of new tissue are generally assumed to be derived from one or more different sources: from division of resident stem cells, by dedifferentiation and subsequent division of differentiated cells, via transdifferentiation of pre-existing cell types (either in the presence or absence of proliferation), or from cells that migrate from sites distant to the wound [2]. In annelids, cell migration from sites distant to the wound have been reported, although these studies have not used lineage tracing methods to track cells in live preparations [26–35]. Currently, we do not know the source of dividing cells in the blastema.

We quantified cell proliferation in pre-existing segments closest to the amputation site, and show there are statistically significant differences in the number of dividing cells in the three segments closest to the wound site compared to the corresponding segments in uncut controls. These differences change over time. Following amputation at the boundary of segment 10 and 11, our data shows that by Stage I (approximately 24 hours p.a.), cell proliferation significantly decreases in the three segments proximal to the cut site (segment 8, 9 and 10; see Results and S1 Fig). It is not currently known whether this shutdown of cell proliferation extends to include other regions of the body, or how rapidly following amputation this phenomenon occurs. A recent study in the naid annelid *Pristina leidyi*, reports a whole body shut down of cell proliferation within 30 minutes of amputation, that persists for several days [23]. The authors propose that long-range signals produced by the wound drive cessation of cell division, perhaps due to an increased need for resources at the expense of other processes dependent on dividing cells. In contrast to the persistent low levels of proliferation in *Pristina*, cell proliferation in *C*. *teleta* recovers to levels comparable to uncut controls by Stage II of regeneration (approximately 48 hours p.a.), concomitant with formation of a morphologically distinguishable blastema. Subsequently, the proportion of dividing cells in segments 9 and 10 significantly increases compared to uncut controls during Stage III and IV. At Stage IV, the proportion of dividing cells in segment 8 is also significantly greater. It is not currently known when (or indeed if) this increased number of proliferating cells return to pre-amputation levels. The proliferating cells within these segments may be a source of cells for the blastema, and could either be born within these segments, or migrate from a distant site.

By Stage V, addition of segments occurs via a posterior growth zone, similar to the addition of segments during growth. However, the zone of proliferating cells in the posterior end of regenerating individuals is broader along the anterior-posterior axis relative to the posterior end of uncut individuals (compare Fig 1A’ and 1G’). This is most likely due to rapid segment addition and growth in these new segments following transverse amputation. In *Platynereis dumerilii*, addition of segments occurs much faster after caudal amputation than during posterior growth. During growth, one posterior segment is added approximately every five days, whereas more than two segments per day are added during post-regeneration growth [25,36,37].

### Regeneration of the ventral nerve cord

We analyzed regeneration of the ventral nervous system and associated peripheral neurons using an anti α-acetylated tubulin antibody to label neurites. At all stages of blastemal outgrowth, neurites extend from pre-existing segments into the blastema, such that contact is always maintained with the posterior end of the newly forming tissue. Initially, the neurites form a web-like pattern that cover the surface of the blastema. Over time neurites gradually become more organized into five longitudinal nerves that straddle the midline. This is the same organization of longitudinal nerves present at the posterior end of uncut animals. By Stage V, multiple new ganglia appear along these longitudinal nerves, and peripheral nerves are also apparent.

The presence of neurites that innervate the wound epidermis has long been known in annelids from classical morphological studies (see [29]), and from more recent studies that utilize fluorescent markers of cell bodies and axons [22,38–42]. Innervation of the blastema during posterior regeneration occurs during a similar timeframe in different annelid species. For example, as in *C*. *teleta*, in *Platynereis dumerilii*, the first indications of nerve fiber regeneration are visible within 2 days post-amputation, and by the following day, innervated anal cirri buds have formed in *P*. *dumerilii*. Intersegmental nerves of the peripheral nervous system are visible within 5 days post-amputation [42], and in *C*. *teleta*, intersegmental nerves appear between Stage IV and V (approximately 5 days and 7 days post-amputation, respectively). Similarly in the oligochaete, *Enchytraeus japonensis*, nerve fibers begin to expand posteriorly from the remaining ventral nerve cord by approximately 3 days post-amputation, and by 5 days post-amputation, a network around the new pygidium has formed [38].

In contrast to similarities in timing of neurite expansion into the blastema, there appear to be differences in the origin of these neurites. In *C*. *teleta*, neurites extend from both the ventral nerve cord and the peripheral nerves near the wound site (Fig 2F”, white, blue and red arrows). Similarly, in the oligochaetes *Enchytraeus fragmentosus*, *Stylaria lacustris*, and *Pristina leidyi*, the nerve network that covers the blastema partly originates from the peripheral nervous system [22,41]. In contrast, in the closely related *E*. *japonensis*, neurites extend from the ventral nerve cord only [38], suggesting that in different species, regenerating neurites are recruited from different sources.

There is evidence that regeneration might depend on the presence of neurons. In 1902, T.H. Morgan described the inhibition of regeneration in the denervated adult annelid *Allolobophora foetida* [43]. Since then, many other annelids have been shown to require nerves to regenerate [29,35,44,45]. Nerve dependence has also been shown for many other invertebrate and vertebrate species (reviewed in [46]). In vertebrates, it is thought that secreted factors from nerves activate cell proliferation and differentiation in the blastema [47]. While this may be the case in annelids [35,45,48], it is intriguing to consider that nerves may also act as “highways” for the migration of neoblasts (annelid stem cells) to the wound site, as has previously been suggested [28,49].

The rapid appearance of 6–8 new ganglia with segmentally-iterated peripheral nerves between Stage IV and Stage V, suggests that multiple new ganglia and segments form simultaneously during regeneration. This has been previously observed in other regenerating annelids [40]. However, examination of a finer time scale between Stage IV and Stage V is required to reveal if there is a currently unappreciated anterior to posterior progression of regenerating peripheral nerves. If there is no anterior to posterior temporal progression, this would represent an example of a key difference between regeneration and development. During development of *C*. *teleta*, the segmentally-iterated peripheral nerves and ganglia form with an anterior to posterior progression [17].

### Most *Hox* genes are expressed in the regeneration blastema

We investigated expression of *C*. *teleta Hox* genes at multiple time points following amputation, during the formation and outgrowth of the blastema. Of the ten *Hox* genes surveyed, seven are expressed in the new tissue between Stage II and Stage V of regeneration. However, the onset of *Hox* gene expression within the regenerating tissue does not follow any temporal co-linearity. That is, genes at one end of the *Hox* cluster are not expressed earlier in regeneration than those at the opposing end of the cluster (Fig 3, genomic organization to the left of panels; see Fig 1A and S3 Fig). In addition, there is no evidence for spatial co-linearity of *Hox* gene expression within the blastema, since there is no simple correspondence between expression boundaries of the *Hox* genes and their relative position within the *Hox* cluster (see Fig 1A and S3 Fig). This result contrasts with *Hox* gene expression patterns during development; *Hox* genes positioned at one end of the genomic cluster are expressed earlier and more anteriorly in *C*. *teleta* larvae than genes located at the other end of the cluster [19]. Thus, *Hox* gene expression patterns in the blastema are not consistent with a role in patterning the anterior-posterior axis of the blastema, and likely do not specify the identity of new segments being formed. Interestingly however, *Hox* genes are expressed in the ventral ectoderm of the blastema in repeated patterns (Stage IV; for example, see Fig 3N, 3S and 3HH), before the appearance of ganglia and peripheral nerves (Stage V). This suggests that the *Hox* genes may have roles in early patterning events or differentiation of cellular subtypes in newly forming segments.

Dividing cells are present at all stages of blastemal outgrowth, and *Hox* genes are expressed during this same timeframe. Proliferating cells first appear in the blastema at Stage II, the same time *CapI-Hox3* expression is initiated. Proliferating cells are distributed throughout the blastema at Stage III and IV, when expression of six more *Hox* genes is detected. The spatial expression patterns of *Hox* genes do not precisely mirror the pattern of EdU-positive cells for any of the *Hox* genes expressed in the blastema, and therefore *Hox* genes are not expressed exclusively in dividing cells. It is likely that *Hox* genes are expressed in a subset of dividing cells (see also Fig 5 and Discussion section, “CapI-Hox3 demarcates a subdomain within the blastema and posterior growth zone). *Hox* genes have been shown to regulate proliferation in multiple cell types in other animals including in *Drosophila* neuroblasts [50], in the vulva of *C*. *elegans* [51] in human breast cancer cells [52], and in mouse neural crest cells [53]. Expression of *Hox* genes in a subset of proliferating cells in the blastema of *C*. *teleta* is consistent with the possibility that they are involved in controlling proliferation during regeneration. Whether they act to promote or inhibit proliferation would require knock down of each *Hox* gene followed by analysis of the number of dividing cells at multiple time points during regeneration, and comparisons to wild-type controls.

There are a number of genes expressed in the regeneration blastema of annelids [14,37,42,54–56]. Thus far, it has been observed that genes expressed in the blastema are also expressed in the PGZ, indicating a link between regeneration and segment addition [57]. However, we show that of the seven *Hox* genes expressed during outgrowth of the blastema, only three are also expressed in the PGZ of uncut juveniles (*CapI-Hox3*, *CapI-Dfd* and *CapI-lox5*), suggesting that at least in *C*. *teleta*, the process of regeneration and segment addition may not be closely linked. Unlike their dynamic expression in the blastema during early stages of regeneration (Stages II-IV), by Stage V when new segments appear, the expression of each *Hox* gene closely resembles that seen in uncut controls. One exception is *CapI-Dfd*, which is expressed in a broader expression domain at Stage V than in uncut controls (Fig 3K and 3O). This broad expression domain may reflect the function of *CapI-Dfd*. For example, although new segments and mature ganglia have formed, *CapI-Dfd* may be required for the differentiation of late forming cell types. Presumably the expression domain of *CapI-Dfd* eventually returns to that of uncut controls.

### *CapI-Hox3* demarcates a subdomain within the blastema and posterior growth zone

To more precisely define the spatial position of *CapI-Hox3* within the posterior growth zone, we combined EdU incorporation and *in situ* hybridization in uncut and regenerating juveniles, using *CapI-Hox3* as a probe. Surprisingly, following a one hour EdU pulse in uncut animals, *CapI-Hox3* is expressed in a heretofore unknown region of the PGZ, which contains a low density of dividing cells. This domain lies directly anterior to the pygidium and posterior to a high density of proliferating cells (Fig 5A–5A”). This result is our first observation of molecular and cellular differences within the PGZ of *C*. *teleta* that define distinct subdomains. During early stages of regeneration (Stage II-III; Fig 5B–5C”), *CapI-Hox3* is expressed in the blastema posterior to the wound site, within the field of dividing cells. At later stages of regeneration (Stage IV-V; Fig 5D–5E”), *CapI-Hox3* is expressed in the PGZ, in an area with a low density of dividing cells, reminiscent of its expression in uncut juveniles.

The expression of *CapI-Hox3* in the posterior end of uncut juveniles is similar to the expression reported for *Hox3* orthologs in juvenile stages of the annelids *Platynereis dumerilii* (*Pdu-hox3*; [56]), and *Alitta virens* (*Nvi-Hox3*; [54]). In both species, *Hox3* is expressed in the segment addition zone (equivalent to the PGZ in *C*. *teleta*), between the pygidium and the most posterior segment. The *Pdu-hox3* expression domain in *Platynereis* includes ectodermal teloblast-like stem cells, thought to contribute to new segments during posterior segment addition [56]. BrdU pulse-chase experiments suggest that these cells divide synchronously and more slowly than adjacent anterior cells [37]. The *CapI-Hox3* expressing cells in *C*. *teleta* may have similar characteristics. As we failed to observe EdU incorporation after a short exposure of one hour in most *CapI-Hox3* expressing cells, we hypothesized that they are simply slowly dividing cells. However, a longer pulse of EdU (eight hours) revealed a similar result, and does not support the hypothesis that the *CapI-Hox3*-expressing cells are synchronously dividing.

The expression of *CapI-Hox3* in the posterior end of uncut juveniles is broadly similar to the expression reported for *Hox3* orthologs in juvenile stages of the annelids *Platynereis dumerilii* (*Pdu-hox3*; [56], and *Alitta virens* (*Nvi-Hox3*; [54]). In both species, *Hox3* is expressed in the segment addition zone (equivalent to the PGZ in *C*. *teleta*), between the pygidium and the most posterior segment. The *Pdu-hox3* expression domain in *Platynereis* includes ectodermal teloblast-like stem cells, thought to contribute to new segments during posterior segment addition [56]. BrdU pulse-chase experiments suggest that these cells divide synchronously and more slowly than adjacent anterior cells [37]. In *C*. *teleta*, for both the one and eight hour pulses of EdU, there are only a few EdU-positive cells within the *CapI-Hox3* expression domain, and the longer pulse of EdU failed to reveal a pattern of synchronous cell divisions. Although one cannot eliminate the possibility that the *CapI-Hox3* expression domain contains a quiescent stem cell population, the current data suggest that there are differences between species in the characteristics of the *Hox3*-expressing cells in the PGZ.

The expression of *CapI-Hox3* in *C*. *teleta* juveniles is a departure from the spatial co-linearity observed for the other genes in the *Hox* cluster, and suggests that *CapI-Hox3* does not play a role in anterior-posterior patterning (see Fig 1B). Larval expression of *Hox3* from the basally branching annelid, *Chaetopterus sp*., has been interpreted as evidence that *Hox3* plays a role in axial patterning in this species [58]. However, the expression patterns of the *Hox3* orthologs in the polychaetes *P*. *dumerilii* and *A*. *virens* resembles that observed in *C*. *teleta*, and suggests that *Hox3* acquired a novel function within annelids. The loss of an axial patterning function for *Hox3* likely occurred prior to the split of the Errantia from the Sedentaria, but following the split of the Chaetopteridae [59]. Interestingly, the loss of a segmental identity function of *Hox3* occurred at least one other time in protostome evolution; in insects the *Hox3* ortholog *zen*, does not specify segmental identity, but rather has roles in development of extraembryonic tissue [60,61].

### Stability of *Hox* gene expression during growth

We previously described the expression of each of the *C*. *teleta Hox* genes during larval development and in 3 day post-metamorphic juveniles [19]. Comparison of expression patterns in 3 day old juveniles (approximately 14–18 segments) from the previous study, with *Hox* gene expression in uncut 2 week post-metamorphic juveniles (approximately 22–28 segments) from the current study, allowed us to draw conclusions regarding the expression of *Hox* genes during growth (i.e. posterior segment addition). During growth, the anterior and posterior expression boundaries of each *Hox* gene remains stable, even as posterior segments are added. For example, *CapI-Antp* is expressed in the ganglia of segments 5–8 in both 3 day and 2 week post-metamorphic juveniles. In another example, *CapI-lox2* is expressed from segment 10 to the final posterior segment (segment 15) in 3 day post-metamorphic juveniles, and is expressed from the ganglia of segment 10 to the final posterior segment in 2 week post-metamorphic juveniles (Fig 4GG shows an animal of 22 segments in length).

However, there are some differences between the expression reported in 3 day post-metamorphic juveniles and in 2 week post-metamorphic juveniles. At 3 days post-metamorphosis, the only *Hox* gene reported to be expressed in the PGZ was *CapI-Hox3*, but in the present study the three genes, *CapI-Hox3*, *CapI-Dfd* and *CapI-lox5*, are expressed in the PGZ of 2 week old juveniles. We believe these differences in expression are due to nutritional status differences rather than due to growth or segment addition. In the previous study, animals were starved for at least 24 hours prior to fixation, while the present study denied animals food for a maximum of 6 hours (see Materials and Methods, “Animal husbandry and amputations”). We have recently become aware of the close relationship between nutritional status, cell division and gene expression levels in juveniles. Nutritional status has been shown to affect growth patterns in other annelids [23]. *C*. *teleta* continuously feeds as a juvenile, and there is a significant decrease in the number of dividing cells in the PGZ within 1–2 days of food removal (data not shown). Each of the three genes expressed in the PGZ at 2 weeks post-metamorphosis also showed expression in the PGZ when their patterns were re-analyzed in unstarved 3 day post-metamorphic juveniles (data not shown).

One other difference between the present study and the results reported in Frobius et al. (2008), is the position of the anterior expression boundary of *CapI-Post2*. Previously it was reported that the anterior boundary of *CapI-Post2* in 3 day post-metamorphic juveniles is fixed at segment 10; however, data collected in this study shows that the anterior boundary is variable among individuals (see Results “Stable expression of Hox genes in pre-existing segments following amputation at the segment 10/11 boundary” and S5 Fig). Re-analysis of the expression of *CapI-Post2* in 3 day post-metamorphic juveniles revealed a variable anterior boundary of expression also. In all cases for 3 day and 2 week old juveniles, the anterior-most segment in which *CapI-Post2* is expressed is the ganglion of segment 10 (the first abdominal ganglia). Our results may differ from the results previously reported due to larger sample sizes analyzed in this study. In total, we analyzed expression for 55 individuals over four independent experiments (S5 Fig). In 3 day post-metamorphic juveniles, the anterior expression boundary differed by as many as 4 segments, while in 2 week juveniles the anterior expression boundary differed by as many as 11 segments among individuals. It should be noted that 3 day post-metamorphic juveniles have only 5–9 abdominal segments, and 2 week post-metamorphic juveniles have 13–19 abdominal segments. Older juveniles may have greater flexibility in the extent to which their anterior expression boundary can vary, since there are more abdominal segments present, and *CapI-Post2* is expressed in abdominal segments. It is notable that in *Alitta virens*, most *Hox* genes also exhibit variable anterior expression boundaries [54], whereas only *CapI-Post2* exhibits a variable anterior expression boundary in *C*. *teleta*.

### *Hox* gene expression in pre-existing segments following amputation

We previously hypothesized that following amputation, *Hox* gene expression in the remaining segments would be re-organized to account for the reduced number of segments of the animal. That is, the remaining body would express the complete suite of *Hox* genes, but each gene would have different anterior-posterior expression boundaries when compared to uncut controls. Surprisingly, we found that expression of most *Hox* genes remained stable following amputation at multiple axial positions, suggesting that at least the ventral nerve cord does not undergo substantial re-patterning. However, amputation at different axial positions resulted in an anterior shift of the anterior expression boundary of *CapI-lox4*, *CapI-lox2* and *CapI-Post2*. The occurrence of the shift depends upon the axial position of the cut, and appears to be related to the original expression domain of the gene (see Fig 6, Fig 7 and S4 Fig). *CapI-lox4* is expressed from the ganglia of segment 7 to the posterior end of the animal in uncut animals, and amputation between segments 7 and 8 or 8 and 9 cause the anterior boundary of expression to shift to segment 6. Amputations further posterior (between 9 and 10 or 10 and 11) do not cause the anterior expression boundary to change. *CapI-lox2* is expressed from the ganglia of segment 10 (the first abdominal segment) through all posterior segments in uncut animals. Amputation at the boundary of segment 9 and 10 or 10 and 11 causes a shift in expression to segment 9; however, no shift in expression occurs following cuts between segment 7 and 8 or 8 and 9. Therefore, *CapI-lox4* shifts its anterior expression boundary by one segment when it is amputated one segment posterior of its original anterior expression boundary, while *CapI-lox2* shifts its anterior boundary of expression by one segment when it is amputated one segment anterior or posterior of its original anterior expression boundary. When amputated at the boundary of segment 9 and 10 or 10 and 11, *CapI-Post2* shifts its anterior expression domain to the ganglia of segment 9. *CapI-Post2* has a variable anterior boundary, so this shift encompasses at least one, but possibly more segments. Notably, for *CapI-lox2* and *CapI-Post2*, amputation can cause the anterior expression domain to cross the thoracic/abdominal boundary. This boundary may be significant, as eight unique combinations of *Hox* genes are expressed in the nine thoracic segments, whereas there is a single *Hox* code for all abdominal segments.

Each of the three genes show an axial shift of expression by 24 hours after amputation, and the timing of the shift in expression varies among each of the three *Hox* genes. *CapI-Post2* changes its anterior boundary within 2 hours, *CapI-lox2* within 4 hours and *CapI-lox4* within 16 hours of amputation. Data for *CapI-lox2* and *CapI-Post2* suggests that it can take up to 5 days until expression domains return to resemble uncut controls. Following amputation between segments 10 and 11, the expression of *CapI-lox2* reverts to that of uncut controls between 24 hours and 3 days post-amputation. In contrast, when amputated at the same position, the anterior boundary of *CapI-Post2* remains in the ganglia of segment 9 until Stage IV (5 days post-amputation), when it reverts to that of segment 10 (Fig 4LL–4NN). From Stage IV until the final day of our analyses (Stage V, approximately 7 days p.a.), the anterior boundary of *CapI-Post2* expression remains at segment 10. We do not know if or when a variable anterior expression boundary in abdominal segments is re-instated for *CapI-Post2*.

Due to the fact that we observe only slight shifts in the anterior boundaries for three *Hox* genes, our data provides only limited evidence for neural morphallaxis in *C*. *teleta*. Morphallaxis has been observed in a number of metazoans, and a number of striking examples have come from studies in annelids. Neural morphallaxis has been shown to occur in the annelid *Lumbriculus variegatus*, where regeneration induces re-organization in the nervous system [11,62–64]. Intact animals display anterior-specific (head withdrawal) and posterior-specific (tail-withdrawal) responses that are mediated by distinct neural pathways. Within days following transverse amputation, posterior segments exhibit anterior-specific responses, a change later accompanied by transformation of neural morphology. A similar change is observed in anterior segments, where posterior escape reflexes are adapted following transverse amputation. It is possible that in *C*. *teleta*, transient changes in *Hox* gene expression could induce a structural and/or physiological shift in the nervous system, although we currently have no data to support this. Whether morphallaxis occurs in other tissues in *C*. *teleta* is currently unknown, although there are precedents for remodeling of the gut in the oligochaetes *Enchytraeus japonensis* [6,55] and *Pristina leidyi* [22].

### *Hox* genes and regeneration across animals

*Hox* genes are involved in regeneration in many animals, including in regeneration of human bone [65], vertebrate limb and tail [66–74], zebrafish tail fin [75], echinoderm arm [76] and planarian posterior body [77,78]. Two recent studies in annelids characterized *Hox* gene expression during regeneration, in the nereid polychaetes *Platynereis dumerilii* [42] and *Alitta virens* (formerly *Nereis virens*) [54].

There are broad similarities among the expression patterns of the *Hox* genes in *A*. *virens*, *P*. *dumerilii* and *C*. *teleta*. For example, in both the nereids and in *C*. *teleta*, most *Hox* genes are expressed in the ganglia of mature segments, and in the blastema during regeneration. In *A*. *virens*, nine out of the ten *Hox* genes (*Nvi-Lox5*, *Nvi-Lox2*, *Nvi-Post2*, *Nvi-Hox1*, *Nvi-Hox4*, *Nvi-Lox4*, *Nvi-Hox7*, *Nvi-Hox2* and *Nvi-Hox3*) are expressed in the blastema following transverse amputation. In *C*. *teleta*, orthologs of each of these genes are also expressed in the blastema, with the exception of *CapI-lab* (*Nvi-Hox1* ortholog) and *CapI-Antp* (*Nvi-Hox7* ortholog). In *P*. *dumerilii*, seven of the nine identified *Hox* genes are expressed in the blastema following transverse amputation (*Pdu-Hox2*, *Pdu-Hox3*, *Pdu-Hox1*, *Pdu-Hox4*, *Pdu-Lox5*, *Pdu-Post2* and *Pdu-Post1*). Orthologs of each of these genes are also expressed in the blastema during *C*. *teleta* regeneration, with the following exceptions: while *Pdu-Hox1* is expressed in the blastema, the *C*.*teleta* ortholog *CapI-lab* is not, and while *CapI-lox2* is expressed in the *C*. *teleta* blastema, the *P*. *dumerilii* ortholog, *Pdu-Lox2* is not. There are some similarities in expression in the blastema between *C*. *teleta*, *A*. *virens* and *P*.*dumerilii*. For example, *Hox3* is expressed in the PGZ in all three species, and *Post2* is broadly expressed early in the blastema in all three species. The fact that most *Hox* genes are expressed in the regeneration blastema for all three species is consistent with the idea that *Hox* genes have an ancestral role in patterning the regeneration blastema of annelids.

Comparisons of *Hox* gene expression patterns in pre-existing tissue between *A*. *virens* and *C*. *teleta* hint at some important functional differences for *Hox* genes following amputation in these two annelids. In *A*. *virens* juveniles, *Hox* genes are expressed in overlapping domains with broad anterior to posterior gradients in the ventral nerve cord [54]. Each gene displays variable anterior boundaries, a fixed boundary at the very posterior of the animal, and are not considered to exhibit spatial or temporal colinearity [54]. Following amputation, there are changes in expression of *Nvi-Lox2*, *Nvi-Post2*, *Nvi-Hox2* and *Nvi-Hox3* in the pre-existing tissue of *A*. *virens*. In each case, amputation causes loss of the entire expression domain, and is followed by re-initiation of expression at the ‘new’ posterior end within 10 hours of amputation. The anterior shift in expression of the *A*. *virens Hox* genes is quite dramatic, and contrasts with the 1–2 segment anterior shifts seen for three *Hox* genes in *C*. *teleta*. For example, in uncut *A*. *virens* juveniles, *Nvi-Post2* is expressed in the four to six posterior-most segments and in the growth zone. Following transverse amputation, *Nvi-Post2* is re-expressed in two to three segments at the new posterior end within four hours of amputation, an anterior shift of approximately ten segments. Similarly, *Nvi-Hox3* is expressed in the growth zone of uncut juveniles, and following removal of the posterior ten segments, it is expressed in the new posterior end within ten hours of amputation. The variable axial position of amputation for *Nvi-Lox2* makes expression data in regenerates difficult to interpret. In intact animals, the anterior boundary of *Nvi-Lox2* expression is at approximately segment 8, and at 4 hours post-amputation, expression is initiated in two pre-existing segments adjacent to the amputation site, indicating a morphallactic shift in expression [54]. However, the specimen shown at 4 hours post-amputation contains 11 segments. This result shows the expected pattern when amputations are conducted at the 11^th^ segment. *C*. *teleta* orthologs of two of these genes, *CapI-lox2* and *CapI-Post2*, show an anterior shift in expression following amputation. Expression patterns in pre-existing segments following amputation have not been analyzed in detail in *P*. *dumerilii*.

The differences in segmental identity between *A*. *virens* and *C*. *teleta* may explain the observed differences in *Hox* gene expression patterns. The body segments in nereids are all very similar to each other morphologically [36]. In *A*. *virens* juveniles, *Hox* genes are expressed in an anterior to posterior gradient, with high levels of expression in the posterior end and variable anterior boundaries. These features have led to the proposal that *Hox* genes function to maintain positional co-ordinates along the anterior-posterior axis in *A*. *virens* [54]. In contrast, *C*. *teleta* has distinct thoracic and abdominal regions, and the *Hox* genes have stable anterior-posterior expression domains, with staggered anterior boundaries. Eight of the nine thoracic segments express a unique combination of *Hox* genes (S4 Fig), although there are only morphological distinctions currently known for a subset of these segments. In the abdomen, three *Hox* genes are expressed in all abdominal segments, regardless of the length of the animal. This suggests that in *C*. *teleta*, most *Hox* genes function to specify segment identity in the thoracic region, while the others maintain positional information in the abdominal region. Notably each of the three *Hox* genes expressed in abdominal segments shift their anterior boundaries of expression following amputation. The variation in *Hox* gene expression patterns among the three annelid species examined thus far emphasizes the challenge in trying to define homologous segments across annelids, and furthermore, highlights the importance of comparative studies within phyla.

## Conclusions

We characterize the contribution of tissue remodeling and formation of new tissue during posterior regeneration in *C*. *teleta*. Posterior regeneration proceeds via epimorphosis and formation of a blastema, and during the first week following transverse amputation, the blastema is populated with numerous proliferating cells. Multiple neurites from pre-existing segments extend into the blastema, and continuously maintain contact with the posterior end of the regenerating tissue. Newly formed ganglia are apparent by approximately 7 days post-amputation (Stage V). Seven *Hox* genes are expressed in the blastema during the first week following amputation, although these genes do not exhibit spatial or temporal colinearity, suggesting regeneration is not a simple recapitulation of development. While the identity of the nervous system is largely stable in pre-existing segments following amputation, we provide the first evidence for limited morphallaxis in the ventral nerve cord based on anterior shifts in expression domains for three *Hox* genes. Future studies examining the effect of amputation on other tissues will reveal whether changes in gene expression in *C*. *teleta* is restricted to neural components, or whether there is an integrated whole body response.

## Supporting Information

S1 Fig Quantification of the ratio of proliferating cells to total nuclei in segments 8, 9 and 10, and in new tissue during regeneration. (A) Schematic representation of areas taken for EdU/nuclei counts. The 8^th^, 9^th^ and 10^th^ segments were defined as the area from the posterior end of the ganglia of the preceding segment to the posterior end of the ganglia in the segment of interest. The 8^th^ segment is shown with a purple square, the 9^th^ segment by a blue square, and the 10^th^ segment by a red square. EdU/nuclei counts for the blastema/new tissue were taken from the posterior end of the ganglia of the last mature segment (segment 10) to the posterior end of the blastema, as indicated by a black square. Ganglia are represented by open horseshoe-shaped structures along the midline of the animal. Double-headed arrows represent pair-wise comparisons between the ratio of EdU-positive nuclei and total nuclei. Comparisons were made between a particular segment (segment 8, 9 or 10) in uncut animals, versus the corresponding segment in regenerating animals. (B) Graphical representation of average EdU-positive nuclei/total nuclei per segment and in the blastema in uncut animals, and at Stage 0, Stage I, Stage II, Stage III, Stage IV and Stage V of regeneration. Each line represents ratios of EdU-positive cells to total nuclei calculated from the 8^th^ (purple), 9^th^ (blue), 10^th^ (red) segment or from blastema/new tissue (black). Error bars represent standard deviations of the mean. Data were generated from at least 5 individuals for each time point.
(TIF)

S2 Fig Reformation of gut ciliation during regeneration. Anti-acetylated α-tubulin showing the ciliated hindgut of *C*. *teleta*. All panels show posterior ends of juveniles in a ventral view, with anterior to the left. Where appropriate, approximate amputation sites are marked with dotted lines, and all tissue to the right of these lines is newly generated tissue. Amputations were conducted at the boundary of segment 10 and 11. All panels are a subset of slices from a z-stack, generated by confocal microscopy. The stage of regeneration is shown to the left of rows. (A) Cilia in the hindgut of uncut animals; (B) The midgut of segments 9 and 10 in an uncut animal does not show gut ciliation; (C) No cilia are visible at Stage II; (D) No cilia are visible at Stage III; (D) Cilia are visible in the new tissue at Stage IV; (E) Cilia are visible in the new tissue at Stage V.
(TIF)

S3 Fig Summary of *Hox* gene expression patterns in the blastema during regeneration. Schematic representation of *Hox* gene expression patterns within the blastema during posterior regeneration of *C*. *teleta*. Posterior ends are depicted as a ventral view, with the dotted lines indicating the amputation site. All tissue to the right of the dotted lines is new tissue. To the left of the figure, a schematic representation of the genomic organization is shown. Black lines depict the two scaffolds containing *Hox* genes, and colored rectangles depict the coding sequences of each *Hox* gene. Rectangles with dotted lines denote genes that are not expressed in the blastema during regeneration (*CapI-lab*, *CapI-Scr* and *CapI-Antp*). The stage of regeneration is shown at the top of the figure.
(TIF)

S4 Fig Summary of *Hox* gene expression in pre-existing tissue during regeneration. Schematic representation of the *Hox* gene expression patterns in the body of *C*.*teleta*, in uncut animals, and at 24 hours following amputation at a specific axial position. At the top of the figure, black lines depict two scaffolds which contain 10 of the *C*. *teleta Hox* genes. Colored rectangles depict the coding sequence of each *Hox* gene. Colored bars indicate expression of *Hox* genes along the anterior-posterior axis. Color coding is the same as that used in the schematic depicting the genomic organization of *Hox* genes. (A) Schematic representation of a juvenile of 15 segments and posterior growth zone (PGZ), with anterior to the left. In uncut animals, the *Hox* genes have defined anterior-posterior boundaries of expression, with staggered anterior boundaries along the main body axis; (B-E) Schematic representation of *Hox* gene expression at 24 hours following amputation at a specific axial position. Transverse amputations were conducted at the boundary of segment 7 and 8 (B), 8 and 9 (C), 9 and 10 (D) and 10 and 11 (E). Black arrows in B, C, D and E indicate instances where an anterior shift in expression has occurred.
(TIF)

S5 Fig Schematic representation of *CapI-Post2* expression pattern in *C*. *teleta* juveniles of different lengths. A total of 55 juveniles of different ages, and with different numbers of segments were used to detect *CapI-Post2* expression by *in situ* hybridization. Segment number is indicated at the top of columns (1–42), with each row representing an individual juvenile (1–55). Blue shading indicates the ganglia in which *CapI-Post2* is expressed, and grey shading indicates segments without *CapI-Post2* expression. A vertical black line between segments 9 and 10 shows the thoracic-abdominal divide.
(TIF)

## Abbreviations

EdU: 5-ethynyl-2-deoxyuridine
FSW: filtered seawater
p.a.: post-amputation
PBS: phosphate-buffered saline
PBT: 1xPBS + 0.1% Triton-X100
PGZ: posterior growth zone
