# Supplementary figures and images for: A Stable Thoracic Hox Code and Epimorphosis Characterize Posterior Regeneration in Capitella teleta
Source: PLoS One. 2016 Feb 19;11(2):e0149724. doi: 10.1371/journal.pone.0149724 (PMC4764619; doi:10.1371/journal.pone.0149724)

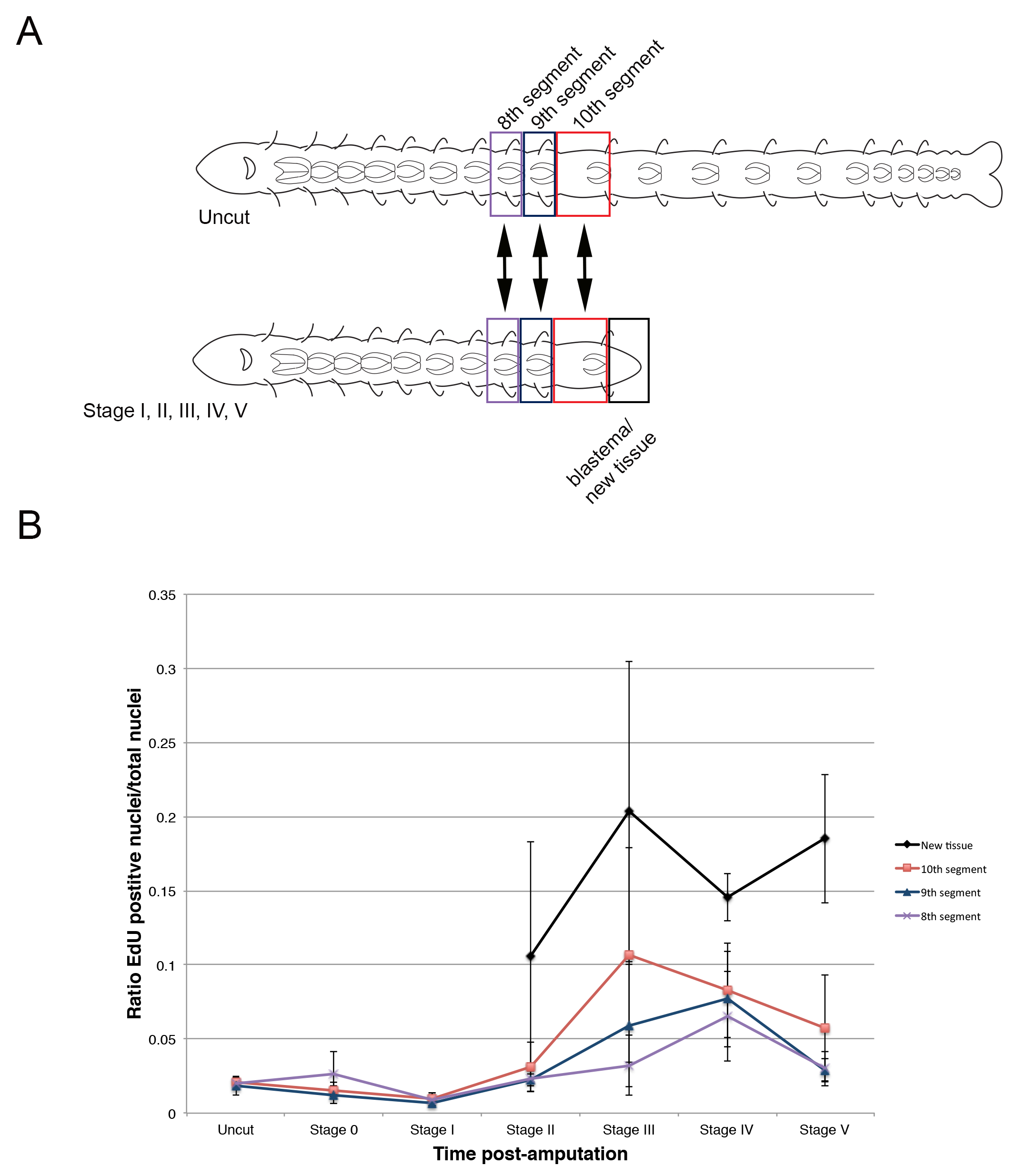

Supplement: S1 Fig — (A) Schematic representation of areas taken for EdU/nuclei counts. The 8th, 9th and 10th segments were defined as the area from the posterior end of the ganglia of the preceding segment to the posterior end of the ganglia in the segment of interest. The 8th segment is shown with a purple square, the 9th segment by a blue square, and the 10th segment by a red square. EdU/nuclei counts for the blastema/new tissue were taken from the posterior end of the ganglia of the last mature segment (segment 10) to the posterior end of the blastema, as indicated by a black square. Ganglia are represented by open horseshoe-shaped structures along the midline of the animal. Double-headed arrows represent pair-wise comparisons between the ratio of EdU-positive nuclei and total nuclei. Comparisons were made between a particular segment (segment 8, 9 or 10) in uncut animals, versus the corresponding segment in regenerating animals. (B) Graphical representation of average EdU-positive nuclei/total nuclei per segment and in the blastema in uncut animals, and at Stage 0, Stage I, Stage II, Stage III, Stage IV and Stage V of regeneration. Each line represents ratios of EdU-positive cells to total nuclei calculated from the 8th (purple), 9th (blue), 10th (red) segment or from blastema/new tissue (black). Error bars represent standard deviations of the mean. Data were generated from at least 5 individuals for each time point. (TIF) [file pone.0149724.s001.tif]

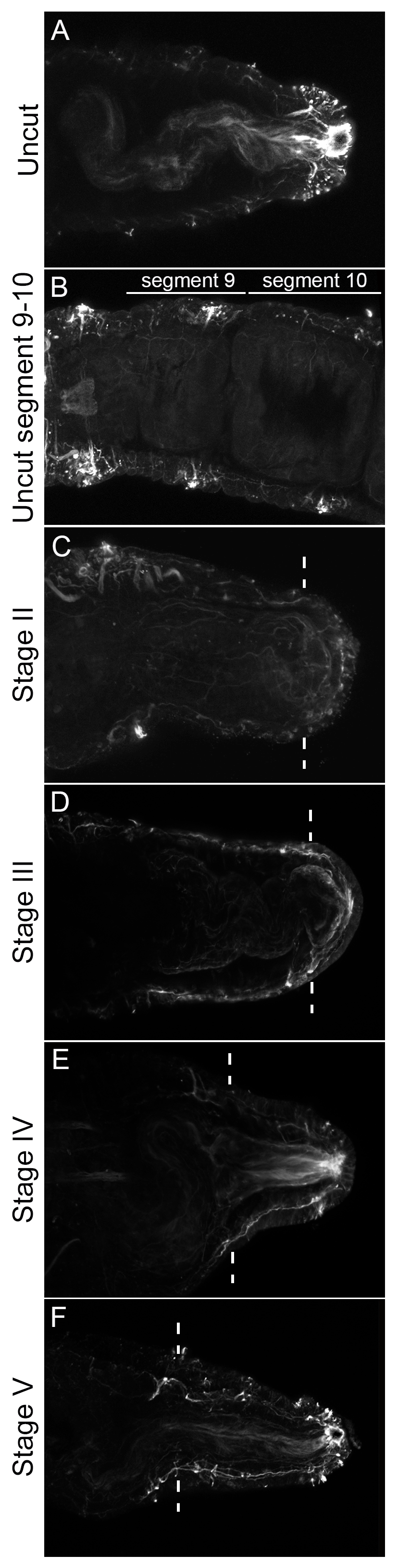

Supplement: S2 Fig — Anti-acetylated α-tubulin showing the ciliated hindgut of C. teleta. All panels show posterior ends of juveniles in a ventral view, with anterior to the left. Where appropriate, approximate amputation sites are marked with dotted lines, and all tissue to the right of these lines is newly generated tissue. Amputations were conducted at the boundary of segment 10 and 11. All panels are a subset of slices from a z-stack, generated by confocal microscopy. The stage of regeneration is shown to the left of rows. (A) Cilia in the hindgut of uncut animals; (B) The midgut of segments 9 and 10 in an uncut animal does not show gut ciliation; (C) No cilia are visible at Stage II; (D) No cilia are visible at Stage III; (D) Cilia are visible in the new tissue at Stage IV; (E) Cilia are visible in the new tissue at Stage V. (TIF) [file pone.0149724.s002.tif]

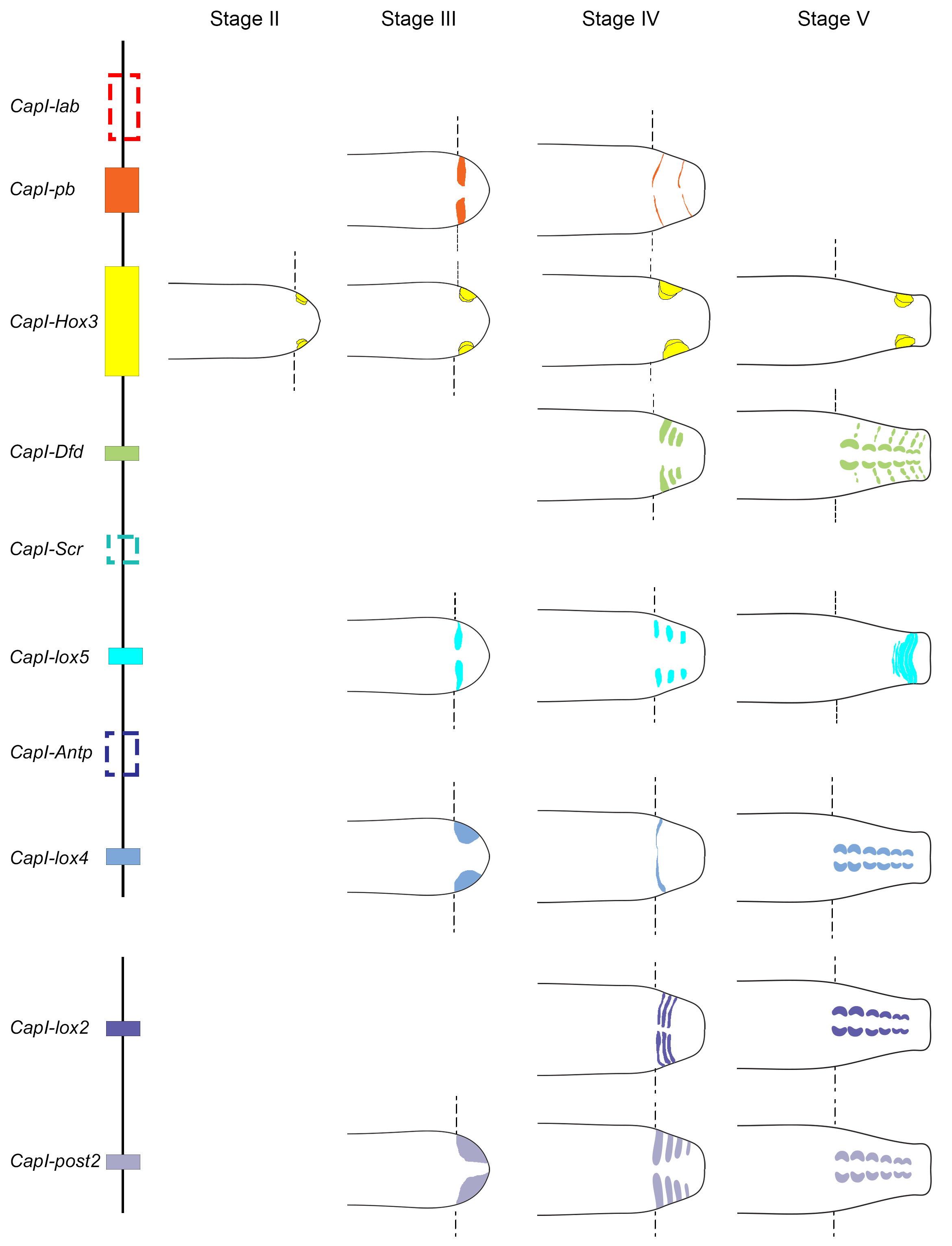

Supplement: S3 Fig — Schematic representation of Hox gene expression patterns within the blastema during posterior regeneration of C. teleta. Posterior ends are depicted as a ventral view, with the dotted lines indicating the amputation site. All tissue to the right of the dotted lines is new tissue. To the left of the figure, a schematic representation of the genomic organization is shown. Black lines depict the two scaffolds containing Hox genes, and colored rectangles depict the coding sequences of each Hox gene. Rectangles with dotted lines denote genes that are not expressed in the blastema during regeneration (CapI-lab, CapI-Scr and CapI-Antp). The stage of regeneration is shown at the top of the figure. (TIF) [file pone.0149724.s003.tif]

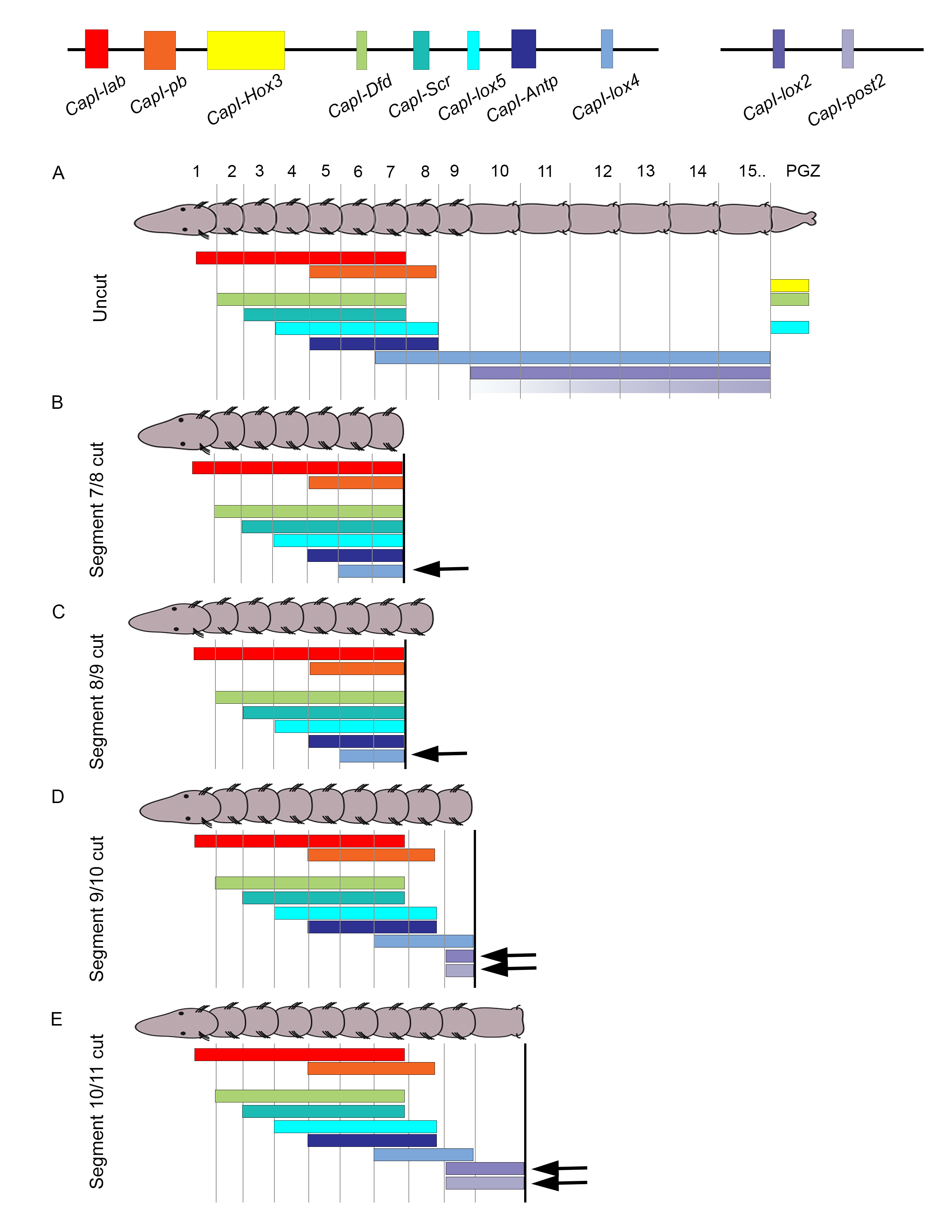

Supplement: S4 Fig — Schematic representation of the Hox gene expression patterns in the body of C.teleta, in uncut animals, and at 24 hours following amputation at a specific axial position. At the top of the figure, black lines depict two scaffolds which contain 10 of the C. teleta Hox genes. Colored rectangles depict the coding sequence of each Hox gene. Colored bars indicate expression of Hox genes along the anterior-posterior axis. Color coding is the same as that used in the schematic depicting the genomic organization of Hox genes. (A) Schematic representation of a juvenile of 15 segments and posterior growth zone (PGZ), with anterior to the left. In uncut animals, the Hox genes have defined anterior-posterior boundaries of expression, with staggered anterior boundaries along the main body axis; (B-E) Schematic representation of Hox gene expression at 24 hours following amputation at a specific axial position. Transverse amputations were conducted at the boundary of segment 7 and 8 (B), 8 and 9 (C), 9 and 10 (D) and 10 and 11 (E). Black arrows in B, C, D and E indicate instances where an anterior shift in expression has occurred. (TIF) [file pone.0149724.s004.tif]

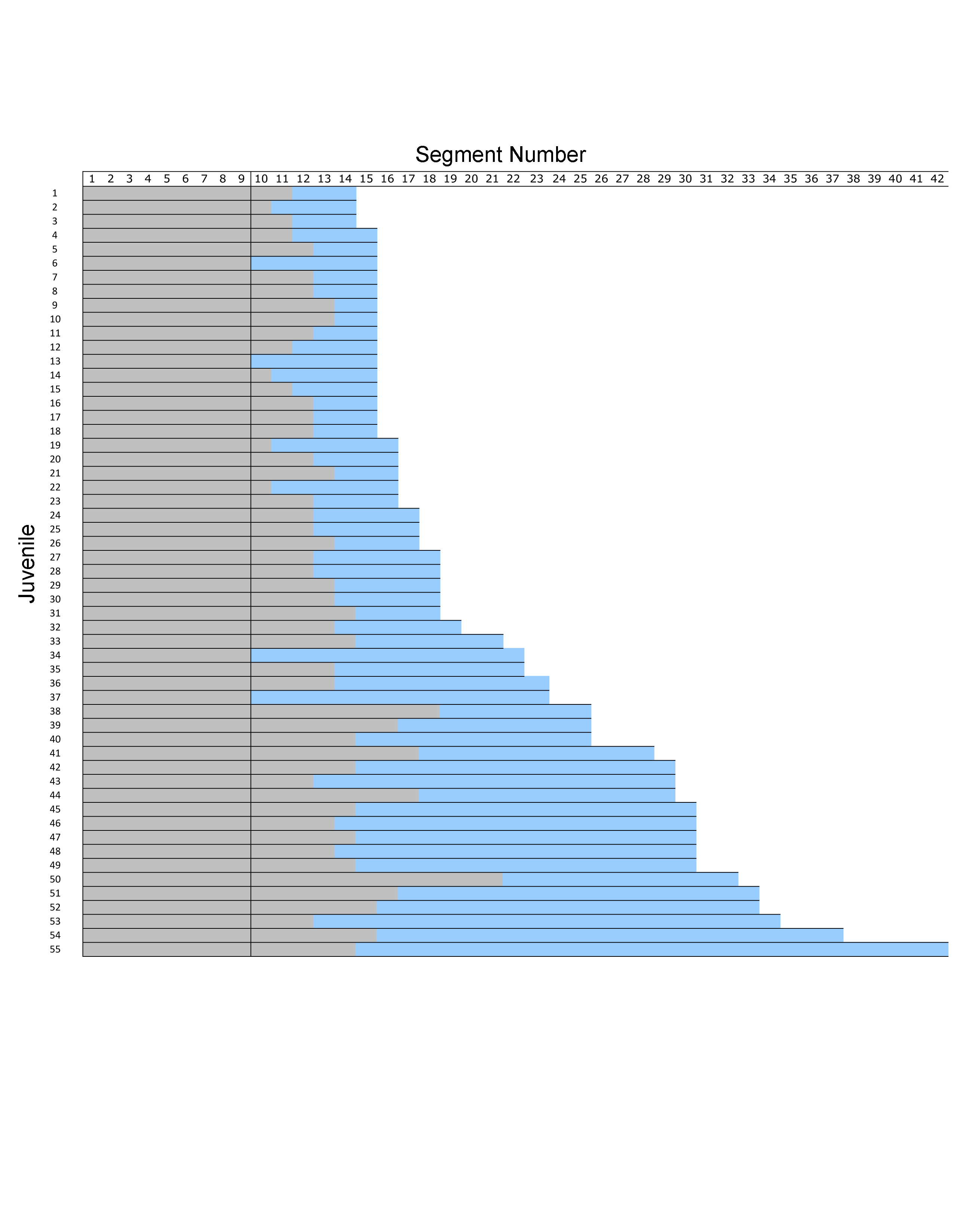

Supplement: S5 Fig — A total of 55 juveniles of different ages, and with different numbers of segments were used to detect CapI-Post2 expression by in situ hybridization. Segment number is indicated at the top of columns (1–42), with each row representing an individual juvenile (1–55). Blue shading indicates the ganglia in which CapI-Post2 is expressed, and grey shading indicates segments without CapI-Post2 expression. A vertical black line between segments 9 and 10 shows the thoracic-abdominal divide. (TIF) [file pone.0149724.s005.tif]
